# The Cooperativity of Atomic Fluctuations in Highly Supercooled Glass-Forming Metallic Melts

**DOI:** 10.1021/acs.jpclett.4c03275

**Published:** 2025-01-21

**Authors:** Jürgen E. K. Schawe, Min Kyung Kwak, Mihai Stoica, Eun Soo Park, Jörg F. Löffler

**Affiliations:** † Laboratory of Metal Physics and Technology, Department of Materials, ETH Zurich, 8093 Zurich, Switzerland; ‡ Department of Materials Science and Engineering, Research Institute of Advanced Materials & Institute of Engineering Research, Seoul National University, Seoul 08826, Republic of Korea

## Abstract

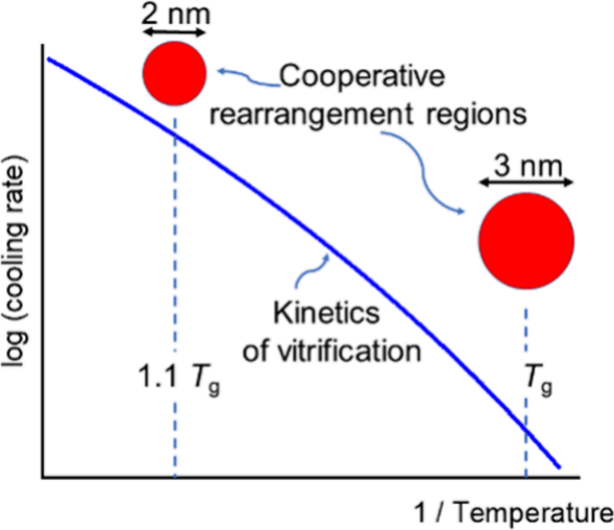

The behavior of supercooled glass-forming metals depends on the cooperative atomic fluctuations caused by dynamic heterogeneities in the melt. These spatial and temporal heterogeneities form dynamic clusters, which are regions of cooperative rearrangement (CRR). In this study, the macroscopic kinetics and the correlation length *ξ*, of the CRR, are derived for Pt_57.4_Cu_14.7_Ni_5.3_P_22.6_ and Pd_43_Cu_27_Ni_10_P_20_ metallic glass-formers by fast differential scanning calorimetry near the glass transition. While the alloy composition influences the α-relaxation and vitrification kinetics, typically defined by the glass transition, as well as the limiting temperature of the Vogel–Fulcher–Tammann–Hesse equation and the fragility index, it has no significant influence on the correlation length of the cooperative atomic motions. In agreement with many other materials, *ξ* is about 3 nm at the glass transition for both metallic glasses. The temperature dependence of *ξ* correlates with the apparent activation energy of the α-relaxation and is the reason for its non-Arrhenius behavior.

A metallic glass is formed if the melt is cooled rapidly enough to prevent crystallization. The critical cooling rate, *β*_c,crit_, is the minimum rate at which such amorphous metal is created. This property can be used to characterize the glass-forming ability (GFA) of a metallic alloy.^1,2^ Glass formation is favored when the atomic mobility in the supercooled liquid is relatively low and the viscosity is high: in glass-forming alloys, the latter is usually about 3 orders of magnitude higher than in pure metals that do not form a glass.^3^ The nanostructure in supercooled glass-forming melts is characterized by the formation of temporally and spatially fluctuating dynamic heterogeneities,^4,5^ which are key to understanding the glass-forming kinetics, and, subsequently, the macroscopic properties of such materials.

Based on von Laue's thermodynamics of fluctuations,^6^ Adam and Gibbs developed the classical theory of supercooled melt in that the dynamic heterogeneities are governed by characteristic thermodynamic subsystems, i.e. cooperative rearrangement regions (CRR) that determine the related configurational entropy.^7^ Many dynamic effects in glass-forming melts can be explained using this classical approach.^8−10^ The macroscopic time–temperature relation of the CRR fluctuations characterizes the α-relaxation. This is the relaxation process associated with the largest change in mechanical properties, where the viscosity changes from liquid to solid.

The dynamics of the α-relaxation in the supercooled melt is studied by using temperature modulated calorimetry and measurement of the vitrification kinetics in a wide cooling rate range on the example of two glass-forming metallic melts with different kinetics but sufficient stability against crystallization. These are the well-known Pt_57.4_Cu_14.7_Ni_5.3_P_22.6_^11^ and Pd_43_Cu_27_Ni_10_P_20_^12^ metallic glasses. From these experimental results the temperature dependence of the correlation length of the CRR is derived using thermodynamic approaches.

The kinetics of α-relaxation can be described as temperature dependence of the average relaxation time *τ*. Below the onset of cooperativity *τ*(*T*) follows the Vogel–Fulcher–Tammann-Hesse (VFTH) equation:^13−15^
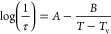
1where *A* and *B* are fitting parameters, and *T*_v_ is the limiting temperature. The relaxation time is related to the measurement frequency according to *τ ω* = 1.

The thermodynamic definition of the glass transition temperature for calorimetric measurements is the limiting fictive temperature *T*_f_ according to Moynihan^16^ and Richardson.^17^ It was used to determine all vitrification temperatures that were measured in the cooling rate range between 0.01 and 10,000 K s^–1^ in Figure 1.

**Figure 1:**
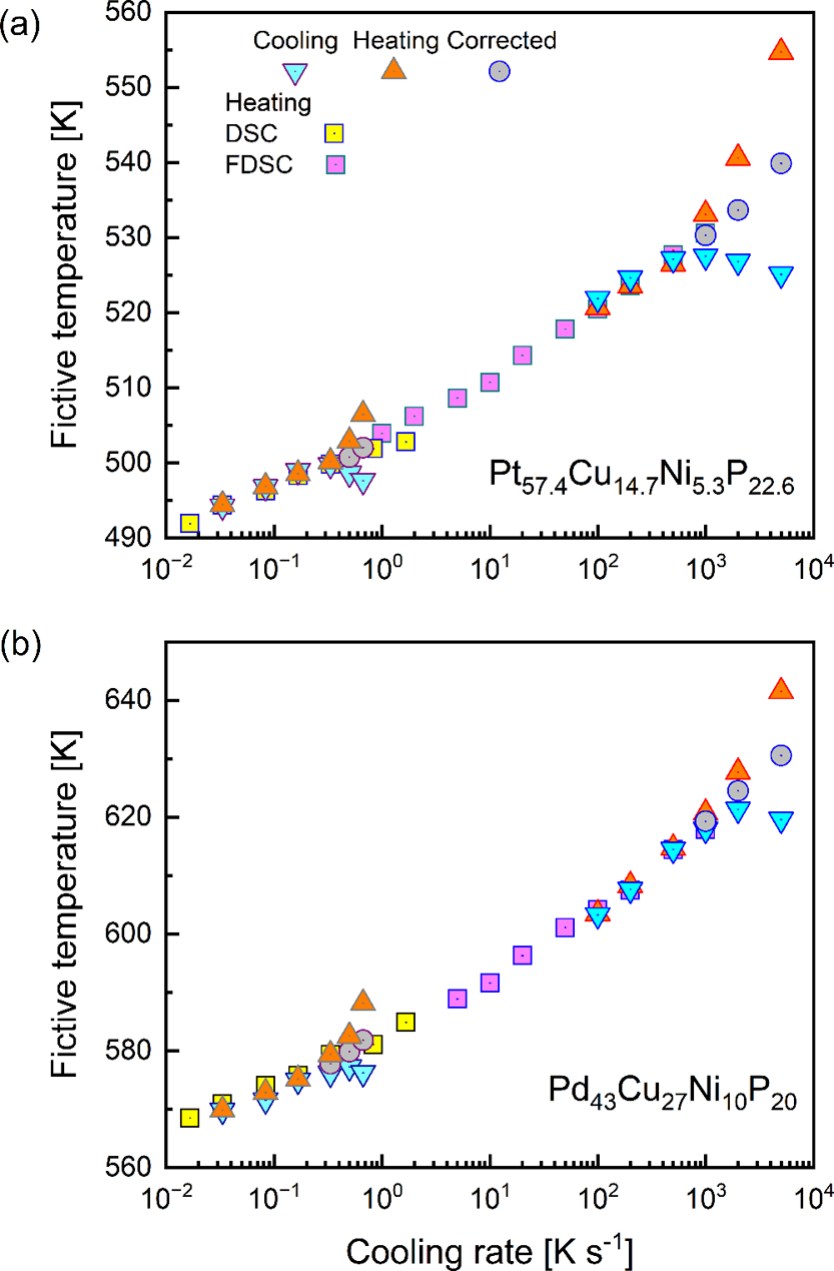
Limiting fictive temperature as a function of the cooling rate or the previous cooling rate for the heating measurements. The triangles are measured upon cooling (downward triangles) and subsequent heating at *β*_h_ = −*β*_c_ (upward triangles), the circles are corrected data, and the squares are data measured at heating with a constant rate of 30 K min^–1^ (yellow squares) or 1000 K s^–1^ (pink squares) after cooling at various rates as indicated by the abscissa. (a) Pt_57.4_Cu_14.7_Ni_5.3_P_22.6_ and (b) Pd_43_Cu_27_Ni_10_P_20_.

For thermo-rheological simple materials, i.e. materials with an almost temperature-invariant relaxation spectrum, the relaxation time is reciprocally proportional to the cooling rate, *β*_c_, at which the material vitrifies at the same temperature:
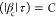
2where *C* is the proportionality constant.^18^ Consequently, the relation between cooling rate and vitrification temperature also follows a VFTH equation with
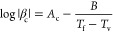
3where *T*_f_ is the fictive temperature, and *B* and *A*_c_ = *A* + log *C* are constants.

Another frequently discussed kinetic parameter of the glass transition is the fragility index, *m*, which was introduced by Angell:^19^
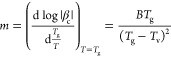
4where *T*_g_ is the glass transition temperature after cooling the liquid at 10 K min^–1^. Minor differences in the fragility index between relaxation and vitrification are due to different definitions of *T*_g_ and/or insufficient consideration of the influence of thermal inertia in the calorimeter.

The cooling rate dependence of *T*_f_ was measured in two ways. First, the samples were cooled at a cooling rate *β*_c_ from the supercooled liquid to the glassy state. Subsequently, they were heated into the melt regime at a heating rate *β*_h_ = |*β*_c_|.^20^ The *T*_f_ determined during heating and cooling must be identical, and deviations are due to uncertainties in determination and thermal inertias of the device and sample during the measurements. Figure 1 shows that the measured *T*_f_ values upon cooling and heating differ at rates above 10 K min^–1^ for conventional DSC and 200 K s^–1^ for FDSC. Above these scanning rates, the measurements must be corrected for thermal lag, for example by using the Tian equation:^21^
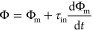
5where Φ and Φ_m_ are the corrected and measured heat flow, and *τ*_in_ is the time constant of the thermal inertia, which is determined from the difference of the measured fictive temperatures of the heating and cooling measurements, Δ*T*_f_ = *T*_f,h_ – *T*_f,c_:
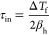
6

The corrected fictive temperature is the mean value between *T*_f,h_ and *T*_f,c_.^18^ These measurements were performed upon heating and cooling with rates between 2 and 40 K min^–1^ for DSC and 100 and 5000 K s^–1^ for FDSC. To close the gap between the two devices, additional measurements were performed using cooling rates between 1 and 50 K min^–1^ (with DSC) and 1 and 500 K s^–1^ (with FDSC). The subsequent heating rate was 30 K min^–1^ (DSC) or 1000 K s^–1^ (FDSC). The fictive temperatures were determined from the heating measurements and corrected according to the thermal inertia. In the respective Arrhenius diagram, the logarithm of the cooling rate is plotted against the reciprocal of the limiting fictive temperature *T*_f_ (Figure 2). The parameters of the VFTH equation are determined by best fit. The glass transition temperature is *T*_f_ at a cooling rate of 10 K/min and the fragility index was calculated from the parameters according to eq 4. These values are listed in Table 1.

**Figure 2:**
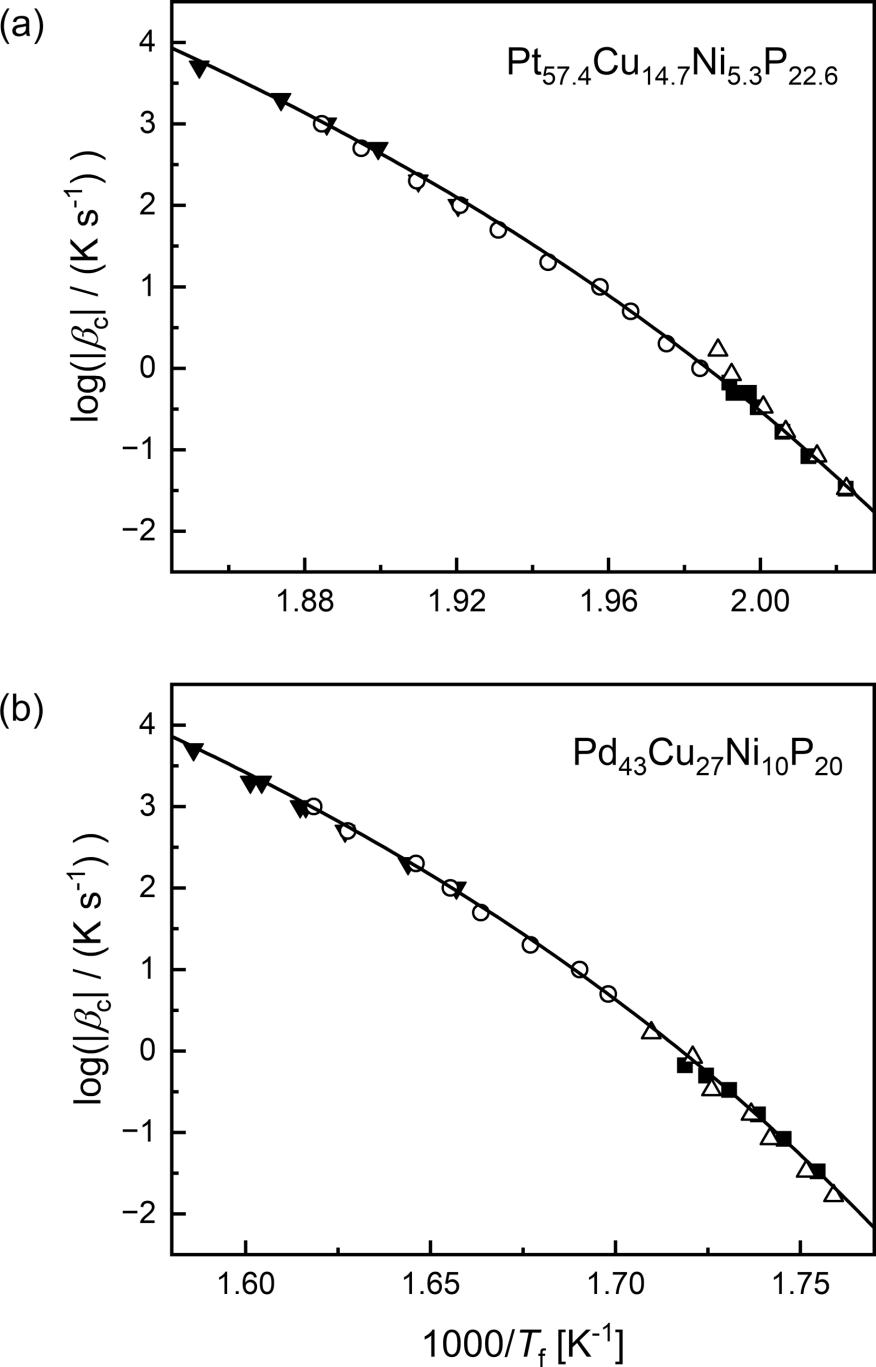
Arrhenius diagrams. The filled symbols are data measured at *β*_c_ = −*β*_h_, while the empty symbols correspond to data measured at a constant heating rate (circles at 1000 K s^–1^ and triangles at 0.5 K s^–1^). (a) Pt_57.4_Cu_14.7_Ni_5.3_P_22.6_, (b) Pd_43_Cu_27_Ni_10_P_20_.

**Table 1:** Characteristic Properties Obtained from the Arrhenius Diagrams in Figure 2

Material	*T*_g_ [K]	*A*_c_	*B* [K]	*T*_v_ [K]	*m*	ξ(*T*_g_) [nm]	[kJ mol^–1^ nm^–1^]
Pt_57.4_Cu_14.7_Ni_5.3_P_22.6_	498	13.7	1310	407.9	80	2.80	376
Pd_43_Cu_27_Ni_10_P_20_	576	12.9	1534	463.2	69	2.65	350

A frequently discussed kinetic parameter is the width of the relaxation spectrum, which describes the distribution of relaxation times. For α-relaxation, the temperature width Δ*T*_ω_, which is measured by modulated calorimetry at the frequency *ω*, is directly related to the width of the relaxation spectrum Δ(ln *ω*).^18^ From
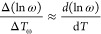
7with *ω* ≈ 1/*τ* we obtain from eq 1 for the relation between the width of temperature dispersion measurements and the corresponding results for frequency dispersion in thermo-rheological simple liquids.
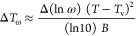
8

The width of the relaxation transition Δ*T*_ω_ was measured using temperature-modulated calorimetry.^22^ The Δ*T*_ω_ were obtained from stochastic modulation (TOPEM) at 10 mHz in DSC and the temperature-modulated FDSC curves at 5 Hz, and correspond to the inflection temperature of the complex heat capacity curve, |*c*_p_*|(*T*).^23^ The width of the vitrification process during cooling, Δ*T*_β_, is larger than Δ*T*_ω_. This effect is described by the vitrification function *κ* = Δ*T*_β_/Δ*T*_ω_,^18^ where *κ* is a constant in the validity range of the VFTH equation.^18,24^ For the investigated materials, *κ* was determined as 2.2 for Pt_57.4_Cu_14.7_Ni_5.3_P_22.6_ and 2.3 for Pd_43_Cu_27_Ni_10_P_20_. Typical cooling cures for Pd_43_Cu_27_Ni_10_P_20_ and the related evaluation of Δ*T*_β_ are shown in Figure 3a. As expected, the temperature width increases with increasing temperature of the α-relaxation.

**Figure 3:**
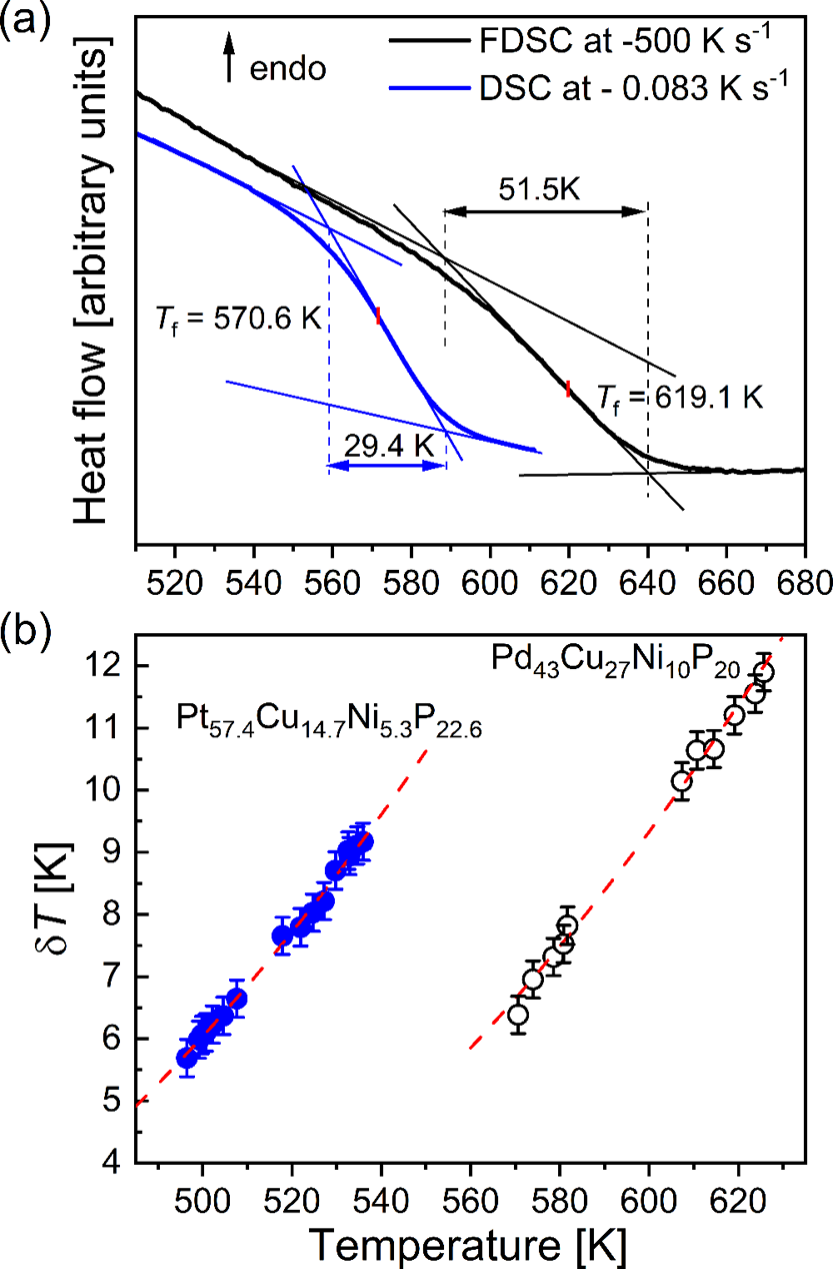
(a) Vitrification curves of Pd_43_Cu_27_Ni_10_P_20_ measured during cooling at −5 K min^–1^ (0.083 K s^–1^) and −500 K s^–1^, and evaluation of the limiting fictive temperature and width Δ*T*_β_. (b) Average temperature fluctuation as a function of temperature. The dashed curves are guides for the eyes.

The macroscopic kinetics of the supercooled melts can be used to analyze the cooperative rearrangements in the melt. The concept of characteristic subsystems with temperature fluctuations in combination with linear response theory to describe the cooperative molecular rearrangement of α-relaxation in supercooled melts has been developed by Donth.^25−27^ This allows to calculate the average characteristic correlation length, *ξ*, of the CRR using the measured thermodynamic data:
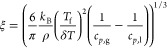
9where *T*_f_ is the characteristic temperature of vitrification, δ*T* is the temperature fluctuation determined from the width of the glass transition, *c*_*p*,g_ and *c*_*p*,l_ are the specific heat capacities of the glassy and liquid state, ρ is the density, and *k*_B_ is the Boltzmann constant. This thermodynamic approach is based on von Laue’s thermodynamics of temperature fluctuations,^6^ which was further developed by Landau and Lifshitz.^28^ The validity of this approach has been recently verified by quasi-elastic neutron scattering for polymeric materials.^29^ The dynamic heterogeneities slightly above the glass transition temperature were visualized by electron correlation spectroscopy using the example of metallic glass nanowires.,^30^ and the result agrees with Donth’s model for CRR.^26^ Furthermore, for organic materials, the correlation lengths determined by dielectric relaxation spectroscopy,^31,32^ NMR,^33^ light scattering^34^ and direct measurements of the configurational entropy at low temperature^35^ agree well with data derived from Donth’s method.^24,36,37^

Although these methods have been applied to nonmetallic glass-forming liquids, Donth’s approach is based on a validated thermodynamic concept without assumptions on themolecular or atomic structure and interaction. We can therefore also safely apply it to atomic systems.

Differential scanning calorimetry (DSC), in conjunction with its fast chip-based version, allows to determine the correlation length over a wide temperature interval by studying vitrification over a large cooling-rate range.^22,24,38^

While it is likely that cooperative spatial fluctuations in highly supercooled metallic melts affect the macroscopic behavior at the glass transition,^39^ such as the mechanical properties^40^ and the kinetics of glass formation and crystallization, there is limited information on the correlation length, *ξ*, and its temperature dependence.^41^ Additionally, the relationship between ξ and the alloy composition remains unclear.

In a recent work,^24^ the influence of structure on the correlation length of the CRR was investigated for two polymers and the chalcogenide glass-former selenium at nearly constant macroscopic kinetics. Here, we compare these results of *ξ*(*T*) with measurements on two metallic glass-formers, which have distinct macroscopic kinetics.

The average temperature fluctuation in the CRR is δ*T*. For slowly crystallizing materials, the temperature dependence of δ*T* can be determined directly from Δ*T*_ω_.^22^ Due to the higher crystallization tendency of metals, a larger temperature range can be achieved by scanning measurements. Therefore, the temperature fluctuation δ*T* was calculated from the vitrification width Δ*T*_β_ of the (corrected) heat flow curves by
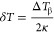
10where the constant *κ* considers the broadening of the transformation interval during vitrification due to the slowing down of the change in relaxation time.^18^Figure 3b reveals that the temperature fluctuation increases with increasing temperature.

The specific heat capacities below and above the glass transition, c_*p*,g_ and c_*p*,l_, were measured using the TOPEM procedure. For the temperatures with known δ*T* (see Figure 3), the correlation length of the CRR was determined using eq 9. The values given in literature were used for the density.^11,12^ The small change in *ρ* with temperature was neglected as it only has a minor influence on the uncertainty of *ξ*. The results are plotted in Figure 4a, and it can be seen that *ξ* increases with decreasing temperature. The relative experimental uncertainty of *ξ* is approximately ±15%. When comparing different methods and different materials, the absolute error is in the order of 0.5 nm. At the glass transition temperature, *ξ* is just below 3 nm for both glass formers. The differences between the two materials of about 0.1 nm correspond to the order of experimental uncertainty. The correlation length is determined in a temperature interval of approximately *T*_g_ ≤ *T* ≤ *T*_g_+50 K. In this range, *ξ* changes by ca. 50%, whereas the cooling rate and consequently the relaxation time (eq 2) changes by about 5 orders of magnitude, as illustrated in Figure 2. These two properties are thus not directly correlated, but it can be assumed that the change in size of the CRR is associated with the non-Arrhenius behavior of the dynamics in the supercooled melts. This can be expressed by an apparent activation energy for the relaxation process, *E*_a_:
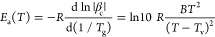
11where *R* is the gas constant. Thus, the apparent activation energy in glass-forming melts increases with decreasing temperature. The concept of thermally activated cooperative rearrangements is widely accepted in literature.^42−44^

**Figure 4:**
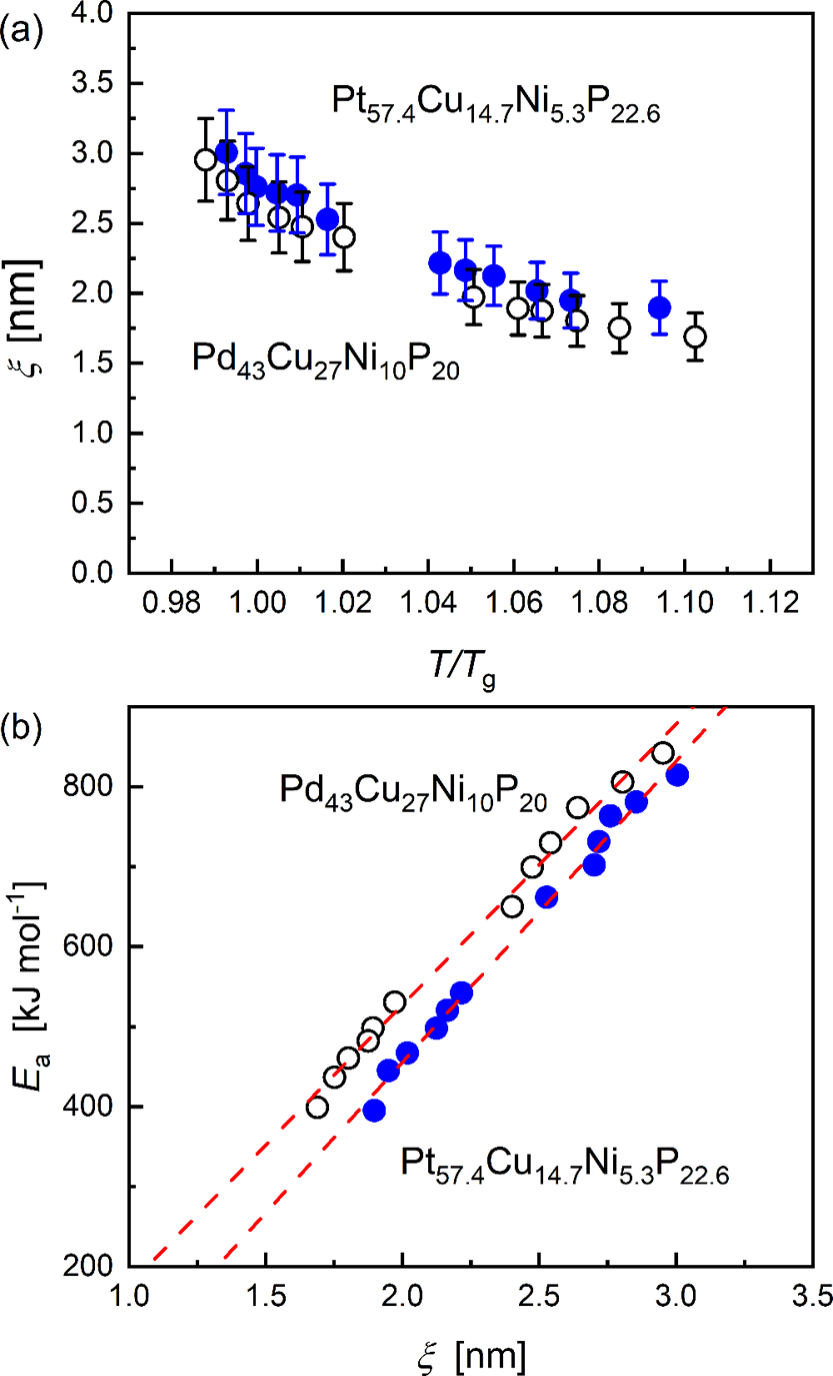
(a) Correlation length of the CRR as a function of reduced temperature *T/T*_*g*_. (b) Apparent activation energy of the α-relaxation as a function of correlation length. The dashed lines are linear fits. The full symbols correspond to Pt_57.4_Cu_14.7_Ni_5.3_P_22.6_ and the open symbols to Pd_43_Cu_27_Ni_10_P_20_.

The comparison of the experimental results of *E*_a_ and ξ in Figure 4b indicates a linear relation, and thus suggests that the curvature in the Arrhenius diagram results from an increase in the size of the CRR. A more extensive discussion of the relation between *E*_a_ and *ξ* is given in the Supporting Information.

The slope  (listed in Table 1) is then a characteristic value that describes the increase in activation energy due to the increase in correlation length of the CRR. This value is expected to correlate with the fragility index *m*. However, the difference between the two slopes in Figure 4b is insignificant for the two alloys investigated. The correlation between the increase of *E*_a_ and the growth of dynamic heterogeneities with temperature decrease, and the insensitivity of the material to the size of dynamic heterogeneities, were also observed in molecular dynamics simulations by the comparison of polymers and metals.^45^

In conclusion, the measured macroscopic kinetics of the metallic melts and their vitrification are consistent with those of low-molecular organic materials and polymers. Therefore, the atomic (or molecular) origins for their macroscopic behavior are most likely comparable for different classes of materials. This is supported by the observation of dynamic heterogeneities in supercooled metallic melts close to vitrification.^30^ By combining conventional DSC and FDSC, and after correcting the influence of thermal inertia, the activation curves of the vitrification process and the correlation length of the cooperative rearrangement regions (CRR) has been determined in a temperature range slightly below *T*_g_ and about 50 K above *T*_g_ for two glass-forming alloys. The temperature range was wide enough to determine the macroscopic kinetic parameters (i.e., the Vogel–Fulcher–Tammann-Hesse (VFTH) parameters *A*, *B* and *T*_v_) and the fragility parameter *m*. The correlation length *ξ* of the CRR increases from about 1.8 nm at *T*/*T*_g_ ≈ 1.1 to ca. 3 nm at *T*_g_. Surprisingly, no significant difference between the two materials has been found for the absolute value of *ξ* and its dependency as a function of reduced temperature *T*/*T*_g_, despite the fact that the kinetic parameters differ largely. This leads us to conclude that the compositional differences between the two glass-forming alloys are relevant for the macroscopic kinetic parameters but less important for the dimensions of the temporary heterogeneities associated with the CRR. The increase in *ξ* with decreasing temperature appears to relate with the change in apparent activation energy of the cooperative atomic movements. From this we can draw three conclusions regarding the composition, the correlation length *ξ*, and the macroscopic kinetics:

(1) The composition and the related atomic interaction strongly influence the VFTH parameters and the glass transition temperature.
(2) The size of the dynamic fluctuations, relevant for the cooperative rearrangements near the glass transition, and its dependency on the reduced temperature *T*/*T*_g_ are not critically influenced by the composition.
(3) The increasing size of the spatial-temporal fluctuating domains with decreasing temperature correlates with the apparent activation energy of the relaxation process. This means that the increase in *ξ* with decreasing temperature is the reason for the non-Arrhenius behavior of the α-relaxation.

## Experimental Methods

Cylindrical ingots of master alloys with a nominal composition of Pd_43_Cu_27_Ni_10_P_20_ and Pt_57.4_Cu_14.7_Ni_5.3_P_22.6_ (at.%)^46^ were acquired from the PX Group, Switzerland. They were cut into small pieces, weighing around 4 g, which were used to prepare amorphous ribbons via the melt spinning technique: the master alloy pieces were inductively melted in quartz crucibles, and then ejected through a nozzle of 1 mm diameter. The casting parameters were kept identical for both alloys. In detail, the nozzle-to-wheel distance was set to 0.3 mm, the ejection overpressure was 300 mbar and the Cu-wheel speed was 20 m/s. Although the melt spinning device is equipped with a two-color pyrometer, the actual ejection temperature could not be measured due to the low melting points of both alloys. The chamber was initially evacuated down to a pressure of 4 × 10^–5^ mbar, and for the melting and melt spinning it was filled with Ar 6.0 purity (99.9999%) up to a pressure of 500 mbar. The melt-spun ribbons were 1 mm wide and around 20 μm thick.

The densities of the two alloys were taken from literature, and are *ρ* = 15.020 g cm^–3^ for Pt_57.4_Cu_14.7_Ni_5.3_P_22.6_^11^ and *ρ* = 9.425 g cm^–3^ for Pd_43_Cu_27_Ni_10_P_20_.^12^

Conventional DSC measurements were performed using a DSC823^e^ Mettler-Toledo device equipped with a sample robot and cryostat. The ribbons were cut into small sections and placed in an oxidized Al crucible. The cell gas was argon and the protective gas was nitrogen. The typical sample size was 60 mg. The temperature range for the Pt-based alloy was between 170 and 275 °C, and for the Pd-based alloy it was between 220 and 350 °C.

Temperature-modulated DSC measurements were performed using the stochastic modulation mode (TOPEM)^47^ of the DSC823^e^ device. The measurements were carried out during cooling with a rate of 1 K min^–1^. The measurement parameters are a temperature amplitude of 1 K and switching times of 30 and 31 s. The evaluation parameters relate to a calculation window of 200 s, a sample response parameter of 20, and an instrumental response parameter of 45. These measurements were performed to determine the heat capacity, and the heat capacity relaxation at 10 mHz.

A Flash DSC 2+ (Mettler-Toledo) with an IntraCooler TC100 (Huber) and a UFH 1 sensor were used for fast DSC (FDSC) measurements. The cell gas was Ar. A thin oil film was prepared on the sample side before inserting the sample with a typical weight of 1 μg. The temperature ranges were 25 °C ≤ *T* ≤ 320 °C and 25 °C ≤ *T* ≤ 440 °C, respectively. Temperature-modulated measurements at 5 Hz were carried out with a sawtooth modulation during cooling from the supercooled melt with an amplitude of 1 K and an underlying cooling rate of 2 K/s (further details in ref (48)).

## Supporting Information Available

Relation between the apparent activation energy *E*_a_ and the correlation length *ξ*; apparent activation energy of the α-relaxation as a function of correlation length (PDF)
Transparent Peer Review report available (PDF)
