# Supplementary material for: The Cooperativity of Atomic Fluctuations in Highly Supercooled Glass-Forming Metallic Melts
Source: J Phys Chem Lett. 2025 Jan 21;16(4):948–54. doi: 10.1021/acs.jpclett.4c03275 (PMC11789148; doi:10.1021/acs.jpclett.4c03275)
Supplement: Supplementary file 1 — jz4c03275_si_001.pdf [file jz4c03275_si_001.pdf]

Supplementary Information

The cooperativity of atomic fluctuations in highly supercooled glass-forming metallic melts

Jürgen E. K. Schawe[1], Min Kyung Kwak[1,2], Mihai Stoica[1], Eun Soo Park[2], Jörg F. Löffler[1]*

[1] Laboratory of Metal Physics and Technology, Department of Materials, ETH Zurich, 8093 Zurich, Switzerland

[2] Department of Materials Science and Engineering, Research Institute of Advanced Materials & Institute of Engineering Research, Seoul National University, Seoul 08826, Republic of Korea

Corresponding Author

*juergen.schawe@mat.ethz.ch

Relation between the apparent activation energy E_a and the correlation length ξ

After solving Eq. (11) for $(T\text{-}T_v)^2$, inserting it into Eq. (8) and considering $\delta T \approx \Delta T_\omega/2$, it follows:

$$\delta T = \frac{R\,\Delta(\ln\omega)\,T^2}{2\,E_a}. \tag{S1}$$

The main contribution to the temperature dependence of ξ in Eq. (9) is the term $T/\delta T$. Thus, this equation can be approximated to

$$\xi \propto \left(\frac{T}{\delta T}\right)^{2/3} \tag{S2}$$

Considering that $\Delta\ln\omega \approx$ const., insertion of Eq. (S1) into Eq. (S2) results in

$$\xi \propto \left(\frac{E_a}{T}\right)^{2/3} \tag{S3}$$

In the measured temperature range, the activation energy varied by 200% and the reciprocal temperature by less than 10%. In the order of experimental uncertainty the term $1/T$ can be assumed to be constant. The exponent is weak and cannot be verified well due to the narrow range in correlation length. Fig. S1 shows Fig. 3b added with the best fit results of Eq. (S3) (black solid lines), resulting in E_a = 161 kJ mol^{-1} nm$^{-3/2}$ $\xi^{3/2}$ for $Pt_{57.4}Cu_{14.7}Ni_{5.3}P_{22.6}$ and E_a = 181 kJ mol^{-1} nm$^{-3/2}$ $\xi^{3/2}$ for $Pd_{43}Cu_{27}Ni_{10}P_{20}$. No significant differences between the linear approximations and fits using Eq. (S3) could be found for the range of measurements. Therefore, we made a linear approximation for simplification.

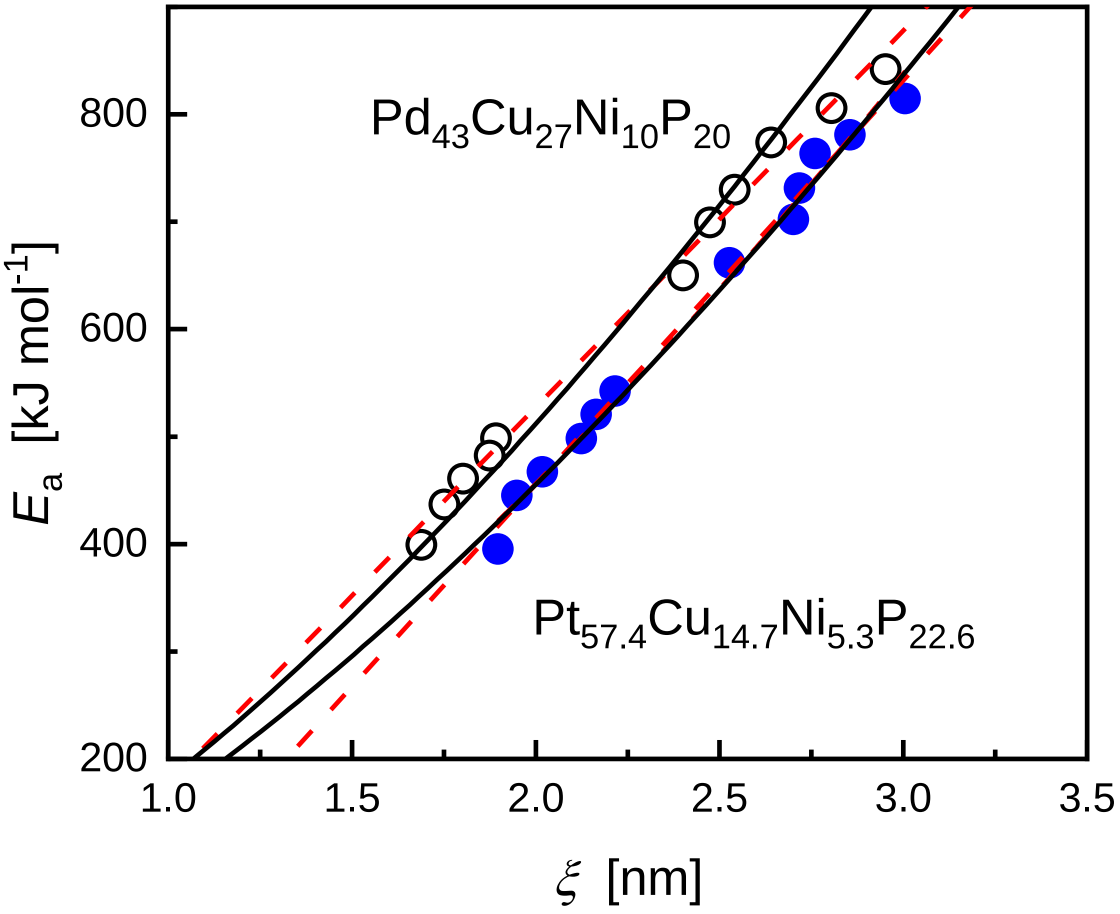

Figure S1. Apparent activation energy of the α-relaxation as a function of correlation length. The full symbols correspond to $Pt_{57.4}Cu_{14.7}Ni_{5.3}P_{22.6}$ and the open symbols to $Pd_{43}Cu_{27}Ni_{10}P_{20}$. The dashed lines are fit results using a linear approximation and the solid lines are results using Eq. (S3).
