# Supplementary material for: The Cooperativity of Atomic Fluctuations in Highly Supercooled Glass-Forming Metallic Melts
Source: J Phys Chem Lett. 2025 Jan 21;16(4):948–54. doi: 10.1021/acs.jpclett.4c03275 (PMC11789148; doi:10.1021/acs.jpclett.4c03275)
Supplement: Supplementary file 2 — jz4c03275_si_002.pdf [file jz4c03275_si_002.pdf]

jz-2024-03275v.R1

Name: Peer Review Information for "The cooperativity of atomic fluctuations in highly supercooled glass-forming metallic melts"

First Round of Reviewer Comments

Reviewer: 1

Comments to the Author

The paper is well-motivated and well-performed, the results are new and highly interesting. I recommend publication in the present form.

Reviewer: 2

Comments to the Author

The authors have measured the vitrification width of two metallic-glass forming liquids in a broad range of cooling rate using the advanced fast scanning calorimetry. While I appreciate the great efforts in conducting such kind of experiments, I have a lot concerns about their argument of measuring size of the cooperative rearrgagement or CRR. This is merely an idea or physical model of Donth that has never been testified. For instance, can the authors provide structural evidence of CRRs in metallic glass, which presumably stored these signatures after vitrification? What is the shape of CRR? Do they have something similar to grain boundaries and how would the boundaries being affected in vitrification? My point is that the so-called correlation length (equation 1) is only an interpretation from the calorimetric results that should not be relied on unless evidence from structural characterization or simulation are provided. It should not serve as the theme of this paper, but could be considered as part of the discussion.

-I must point out there are other possibilities transferring the glass-transition width or delta_T, indluding Sci. Adv. 2020 6 eaay1454 and Ncommun 2023 14 4698. They used delta_T (or something equivalent) dividing the scanning rate to work out a relaxation time, which forms an Angell's plot, fits in VFT and results in fragility index pretty well. What is the difference between calculating correlation length and relaxation time? Are there anything new we can learn from this new parameter? The authors should discuss other possibilities and compare them with their parameter.

-The authors should provide calorimetry curves showing width change with the scanning rate because this is what they measured. They should also label the vitrification width showing us how it is defined.

-Lastly, I don't like Ea or the apparent activation energy for alpha relaxation. It is merely the slope of VFT by definition (Eq. 9), and there are no evidence that alpha relaxation has a broad range of activation energy (technically, it would not be termed as a specific relaxation mode if the activation energy deviates a lot). Besides, no assistance to their argument can be provided by Fig. 4b, which is hardly mentioned.

-In conclusion, I cannot recommend this paper for publication. The authors should rewirte the paper, highlighting the key observations and separating interpretations from their results.

Author's Response to Peer Review Comments:

Response to the Editor and the Reviewers

We would like to thank the reviewers for acknowledging our work and appreciate the reviewers' suggestions, which helped us to improve the manuscript.

Changes have been made as explained in the following and as identified in the highlighted version of the revised manuscript.

Comment to the Editor:

We would like to thank the editor for giving us the opportunity to revise this manuscript for publication.

Response to Reviewers' comments

Reviewer #1

This paper represents a significant new contribution and should be published as is.

Authors:

Thank you! We appreciate this very positive comment.

Reviewer #2

(1) Recommendation: This paper may be publishable, but major revision is needed; I would like to be invited to review any future revision.

Authors:

Thank you for your work and your comments. I am sure that your feedback will help us to clarify our conclusions made in the manuscript.

(2) The authors have measured the vitrification width of two metallic-glass forming liquids in a broad range of cooling rate using the advanced fast scanning calorimetry. While I appreciate the great efforts in conducting such kind of experiments, I have a lot concerns about their argument of measuring size of the cooperative rearrgagement or CRR. This is merely an idea or physical model of Donth that has never been testified.

Authors:

First of all, we would like to thank the reviewer for the appreciation of our experimental work regarding the kinetics of the glass transition in metals.

We appreciate the reviewer's concerns about the approach of measuring the size of CRRs. However, in the last years there has been significant progress in the field.

Although the principles of the method used were developed by Donth more than 40 years ago, we only started using it this year. The reason for this was that for us this method was based on an assumption about thermodynamics that had not been sufficiently confirmed until recently.

However, the recently published paper by R. Zorn et al., *ACS Phys. Chem. Au* (Ref. 13) made us change our mind. In this paper, Donth's method is tested using quasi-elastic neutron scattering. The results show very good agreement of the measured correlation lengths as well as good arguments for the validity of the thermodynamics used by Donth.

A further argument for the use of this approach is the work of Zang P. et al., *Nat. Comm*. **9**, 1129 (2018), in which dynamic heterogeneities in a supercooled metal melt just above the glass transition have been visualized by electron correlation spectroscopy. Despite the reduced resolution of about 1 nm, the data agree well with Donth's predictions and our results.

The third argument in favor of using Donth's method is the good agreement with results obtained with completely different methods such as dielectric relaxation spectroscopy (Cangialosi, D. et al., *J. Chem. Phys.* **128**, 224508 (2008)) and light scattering (Hong, L. et al., *Phys. Rev E* **83**, 061508 (2011)) for organic materials. Unfortunately, these methods cannot be used for metals.

To clarify this point we modified the manuscript and add these references:

Page 9: Further validation for the applicability of this method is based on the visualization of dynamic heterogeneities in supercooled metal melts by electron correlation spectroscopy[25] and their agreement with Donth's model.[22] Furthermore, for organic materials, the correlation lengths determined by dielectric relaxation spectroscopy[26] and light scattering[27] agree well with data derived from Donth's method.[28,29,30]

Page 13: In conclusion, the measured macroscopic kinetics of the metallic melts and their vitrification a consistent with those of low-molecular organic materials and polymers. Therefore, the atomic (or molecular) origins for their macroscopic behavior are most likely comparable for different classes of materials. This is supported by the observation of dynamic heterogeneities in supercooled metallic melts close to vitrification.[25]

Added references:

(25) Zhang, P.; Maldonis, J. J.; Liu, Z.; Schroers, J.; Voyles, P. M. Spatially heterogeneous dynamics in a metallic glass forming liquid imaged by electron correlation microscopy. *Nat. Comm*. **2018**, *9*, 1129.

(26) Cangialosi, D., Alegría, A.; Colmenero, J. Dielectric relaxation of polychlorinated biphenyl/toluenemixtures: Component dynamics. *J. Chem. Phys.* **2008**, *128*, 224508.

(27) Hong, L., Novikov, V.N.; Sokolov, A.P. Dynamic heterogeneities, boson peak, and activation volume in glass-forming liquids. *Phys. Rev E* **2011**, *83*, 061508.

(28) Hempel, E.; Hempel, G.; Hensel, A.; Schick, C.; Donth, E. Characteristic length of dynamic glass transition near Tg for a wide assortment of glass-forming substances. *J. Phys. Chem. B* **2000**, *104*, 2460-2466.

(29) Rijal, B., Delbreilh, L.; Saiter, A. Dynamic Heterogeneity and Cooperative Length Scale at Dynamic Glass Transition in Glass Forming Liquids. *Macromolecules* **2015**, *48*, 8219–8231.

(3) For instance, can the authors provide structural evidence of CRRs in metallic glass, which presumably stored these signatures after vitrification?

Authors:

The characteristic movements associated with CRR occur in the supercooled melt. In the glass, the corresponding configurational heat capacity is not present and no long-range fluctuations occur. Long-range dynamic heterogeneities do not exist in the glass. The only residues are the quasi-static structural heterogeneities, which are the reason for the medium-range order and the low density of the glass. The glass structure is not the subject of the manuscript.

(4) What is the shape of CRR?

Authors:

The CRRs include regions with low and high mobility. The figures in Ref. (13a) give the best available impression of the shape of the CRR.

(5) Do they have something similar to grain boundaries and how would the boundaries being affected in vitrification?

Authors:

Grain boundaries indicate differences in order. In the case of CRR, there are only differences in dynamics. CRR varies in time and space. The “lifetime” of heterogeneities is slightly longer than their characteristic relaxation time. The correlation length is only a spatial distance in which, on average, all possible relaxation times of the long-distance movements can be realized.

(6) My point is that the so-called correlation length (equation 1) is only an interpretation from the calorimetric results that should not be relied on unless evidence from structural characterization or simulation are provided. It should not serve as the theme of this paper, but could be considered as part of the discussion.

Authors:

As mentioned above, dynamic heterogeneities are not only measured by calorimetry. It is only the simplest method with the disadvantage that it does not provide visual results like electron correlation spectroscopy. The advantage is the possibility of covering a wide temperature range with modern calorimetry. With quasi-elastic neutron spectroscopy, it has been shown that the thermodynamics of von Laue and Landau on which the used technique is based on is valid.

To comply with the suggestion of the reviewer we rearranged the manuscript accordingly. This led to revisions in the abstract and on pages 3, 8, 9 and 10. Text modifications are marked in red and the shifted text in green.

(7) -I must point out there are other possibilities transferring the glass-transition width or delta_T, indluding Sci. Adv. 2020 6 eaay1454 and Ncommun 2023 14 4698. They used delta_T (or something equivalent) dividing the scanning rate to work out a relaxation time, which forms an Angell's plot, fits in VFT and results in fragility index pretty well.

Authors:

The first remark regarding the determination of the relaxation time:

The method described is one of the many approximations to obtain a relaxation time from a single DSC measurement. We have never seen a discussion of the associated experimental error

and scientific explanation in the literature regarding the method mentioned by the reviewer. In contrast, we have doubts about this method due to the broadening of the glass transition interval with increased cooling rate on the one hand, and the experimental verification of the inverse proportionality between cooling rate and averaged relaxation time on the other.

We prefer a different technique, which was discussed and verified in detail in Ref. 17. The method used has two advantages. First, it corrects for the influence of thermal inertia on the derived data. This is essential especially for scanning rates of above 5 K/min for conventional DSC and 1000 K/s for FDSC. Secondly, additional relaxation spectra are measured using modulated measurements and the results are used to improve the data.

Angell's plot:

The activation diagram and Angell's diagram are equivalent in this discussion. The only difference is the abscissa used. In the activation diagram the absolute temperature is used, in Angell's diagram the reduced temperature T/T_g. The problem with the latter diagram is the definition of the glass transition temperature. Small deviations influence the shape of the curve. For the activation diagram, one critical parameter less is needed, and the diagram therefore directly shows the measured data, which is available for further discussion and evaluation without additional assumptions. The glass transition temperature is then only required for the determination of the fragility index, whereby the same experimental uncertainties exist as with the Angell's plot.

(8) What is the difference between calculating correlation length and relaxation time?

Authors:

The *averaged* relaxation time (we interpret the question of the reviewer in this way; otherwise we refer to P. Debye (1934) https://doi.org/10.1039/TF9343000679 and J. Frenkel, Kinetic theory of liquids, Oxford Press (1946)) is a single experimental parameter and the correlation length of the dynamic heterogeneities is a contribution to a possible explanation of the measured macroscopic kinetics.

(9) Are there anything new we can learn from this new parameter?

Authors:

The answer to this question is in the conclusions:
Two glass-forming metals with different macroscopic kinetics are used. But the temperature dependence of the correlation lengths is identical within the limits of the experimental uncertainties. A comparison with other glass formers (polymers and chalcogenides) shows that the lengths have comparable values and a comparable behavior towards the reduced temperature. This is an indication that the cooperativity (near the glass transition) is a consistent

parameter for a larger group of glass formers. We have shown this for the first time experimentally for atomic systems.
The linear relationship between the correlation length and the apparent activation energy of relaxation in the supercooled metal melts indicates the reason for the non-Arrhenius behavior of the glass-forming liquids. To our knowledge, this has not yet been demonstrated.

(10) The authors should discuss other possibilities and compare them with their parameter.

Authors:

We do not understand the reviewer on this point. Possibilities for what?

(11) -The authors should provide calorimetry curves showing width change with the scanning rate because this is what they measured. They should also label the vitrification width showing us how it is defined.

Authors:

Thank you for this suggestion. A related graph is added in Fig. 3a. The text is modified accordingly:

Page 10: Typical cooling cures for $Pd_{43}Cu_{27}Ni_{10}P_{20}$ and the related evaluation of ΔT_β are shown in Fig. 3a.

(12) -Lastly, I don't like Ea or the apparent activation energy for alpha relaxation. It is merely the slope of VFT by definition (Eq. 9), and there are no evidence that alpha relaxation has a broad range of activation energy (technically, it would not be termed as a specific relaxation mode if the activation energy deviates a lot). Besides, no assistance to their argument can be provided by Fig. 4b, which is hardly mentioned.

Authors:

We understand this statement, but cannot agree with it without sound scientific arguments. In contrast, the formation of dynamic heterogeneities must be thermally activated. This is also a conclusion of the energy landscape model developed by Goldstein, which is one of the muchdiscussed foundations of modern physical chemistry of supercooled glass-forming liquids and glass formation. In this regard, we would like to ask the reviewer on how the strong nonArrhenius change in the temperature dependence of alpha relaxation compared to the JohariGoldstein relaxation can be explained? There are many arguments that this difference is related to the cooperativity of molecular rearrangements and the size change of the dynamic heterogeneities in the supercooled melt (see e.g. Fig. 4b or reference (37)).

In order to underline that this is not only our individual subjective belief, but a serious part of the current scientific discussion on this topic, we include an article in the reference list as an example.

Page 12: Thus, the apparent activation energy in glass forming melts increases with decreasing temperature.

Page 12: The correlation between the increase of E_a and the growth of dynamic heterogeneities with temperature decrease as well as the insensitivity of the material on the size of dynamic heterogeneities, was also observed in molecular dynamic simulations.[37]

Added reference:

(37) Zhang, H.; Wang, X.; Yu, H.-B.; Douglas, J. F. Dynamic heterogeneity, cooperative motion, and Johari-Goldstein β-relaxation in a metallic glass-forming material exhibiting a fragile-to-strong transition, *Eur. Phys. J. E* **2021**, *44*, 56.

(13) -In conclusion, I cannot recommend this paper for publication. The authors should rewirte the paper, highlighting the key observations and separating interpretations from their results.

Authors:

It is expected that a topic such as the theory of glass formation from supercooled liquids will be controversial. Our intention is to explore the discussion on the origin of glass formation in different materials classes in terms of their specificities.
The conclusion of this work is that we do not find any obvious differences between metals and organic materials in the macroscopic kinetics regarding their atomic/molecular rearrangements. On the contrary we find strong similarities that will open new research avenues. As we consider this result to be important for a broader research community, as suggest by reviewer #1, we have chosen JPCL for publishing these data. In our opinion, the comments and interest of reviewer #2 confirm this impression.

HIGHLIGHTED VERSION OF THE REVISED MANUSCRIPT.

The cooperativity of atomic fluctuations in highly supercooled glass-forming metallic melts

Jürgen E. K. Schawe[1], Min Kyung Kwak[1,1], Mihai Stoica[1], Eun Soo Park[2], Jörg F. Löffler[1]*

[1] Laboratory of Metal Physics and Technology, Department of Materials, ETH Zurich, 8093 Zurich, Switzerland

[1] Department of Materials Science and Engineering, Research Institute of Advanced Materials & Institute of Engineering Research, Seoul National University, Seoul 08826, Republic of Korea

Corresponding Author

*juergen.schawe@mat.ethz.ch

ABSTRACT. The behavior of supercooled glass-forming metals depends on the cooperative atomic fluctuations caused by dynamic heterogeneities in the melt. These spatial and temporal heterogeneities form dynamic clusters, which are regions of cooperative rearrangement (CRR). In this study, the macroscopic kinetics and the correlation length ξ, characteristic of the CRR, is are derived for $Pt_{57.4}Cu_{14.7}Ni_{5.3}P_{22.6}$ and $Pd_{43}Cu_{27}Ni_{10}P_{20}$ metallic glasses glass-formers by fast differential scanning calorimetry at temperatures near the glass transition. While the alloy composition influences the macroscopic α-relaxation and vitrification kinetics, typically defined by the glass transition, as well as the limiting temperature of the Vogel-Fulcher-Tammann-Hesse equation and the fragility index, it has no significant influence on the correlation length of the cooperative atomic motions. In agreement with many other materials, ξ is about 3 nm at the glass transition for both metallic glasses. The temperature dependence of ξ correlates with the apparent activation energy of the α-relaxation and is the reason for their its non-Arrhenius behavior.

TOC GRAPHICS

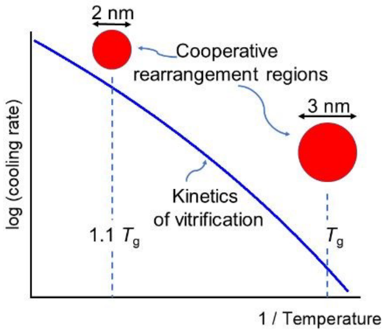

KEYWORDS metallic glasses; glass transition; dynamic heterogeneities; supercooled melt; cooperative rearrangements.

A metallic glass is formed if the melt is cooled rapidly enough to prevent complete crystallization. The critical cooling rate, $\beta_{c,crit}$, is the minimum rate at which such amorphous metal is created. This property can be used to characterize the glass-forming ability (GFA) of a metallic alloy. [1,2] Glass formation is favored when the atomic mobility in the supercooled liquid is relatively low and the viscosity is high: in glass-forming alloys, the latter is usually about three orders of magnitude higher than in pure metals that do not form a glass.[3] The nanostructure in supercooled glass-forming melts is characterized by the formation of temporally and spatially fluctuating dynamic heterogeneities,[4,5] which are key to understanding the glass-forming kinetics, and, subsequently, the macroscopic properties of such materials.

According to the classical thermodynamic theory of supercooled melts by Adam and Gibbs, the dynamic heterogeneities are governed by characteristic thermodynamic subsystems, i.e. cooperative rearrangement regions (CRR) that determine the related configurational entropy.[6] Many dynamic effects

in glass-forming melts can be explained using this classical approach.[7,8,9] The macroscopic timetemperature relation of the CRR fluctuations characterizes the α-relaxation. This is the relaxation process associated with the largest change in mechanical properties, where the viscosity changes from liquid to solid. The dynamics of the glass transition and the temperature dependence of the correlation length of the CRR are studied on the example of two glass-forming metallic melts with different kinetics but sufficient stability against crystallization. These are the well-known $Pt_{57.4}Cu_{14.7}Ni_{5.3}P_{22.6}$ [10] and $Pd_{43}Cu_{27}Ni_{10}P_{20}$.[11]

The kinetics of α-relaxation can be described as temperature dependence of the average relaxation time

τ. Below the onset of cooperativity $\tau(T)$ follows the Vogel-Fulcher-Tammann-Hesse (VFTH)

equation:[12,13,14]

$$\log\left(\frac{1}{\tau}\right) = A - \frac{B}{T - T_v} \quad (1)$$

where A and B are fitting parameters, and T_v is the limiting temperature. The relaxation time is related to the measurement frequency according to $\tau\,\omega = 1$.

The thermodynamic definition of the glass transition temperature for calorimetric measurements is the limiting fictive temperature, T_f, according to Moynihan[15] and Richardson.[16]. It was used to determine all vitrification temperatures that were measured in the cooling rate range between 0.01 and 10,000 K s^{-1} in (Fig. 1).

For thermo-rheological simple materials, i.e. materials with an almost temperature-invariant relaxation spectrum, the relaxation time is reciprocally proportional to the cooling rate, β_c, at which the material vitrifies at the same temperature:

$$(|\beta_c|\,\tau)/1\ \mathrm{K} = C \quad (2)$$

where C is the proportionality constant.[17] Consequently, the relation between cooling rate and vitrification temperature also follows a VFTH equation with:

$$\log|\beta_c| = A_c - \frac{B}{T_f - T_v} \quad (3)$$

where T_f is the fictive temperature, and B and $A_c = A + \log C$ are constants.

Another frequently discussed kinetic parameter of the glass transition is the fragility index, m, which was introduced by Angell:[18]

d $\log|\beta_c|$ $B\,T_g$

$$m = \left(\frac{\quad}{T_g \, \overline{\;T\;}} \right)_{T=T_g} = T_g \quad \frac{\quad}{(T_{v2} \quad)} \quad (4) \; \mathrm{d}$$

where T_g is the glass transition temperature after cooling the liquid at 10 K min^{-1}. Minor differences in the fragility index between relaxation and vitrification are due to different definitions of T_g and/or insufficient consideration of the influence of thermal inertia in the calorimeter.

The cooling rate dependence of T_f was measured in two ways. First, the samples were cooled at a cooling rate β_c from the supercooled liquid to the glassy state. Subsequently, they were heated into the melt regime at a heating rate $\beta_h = |\beta_c|$.[19] The T_f determined during heating and cooling must be identical, and deviations are due to uncertainties in determination and thermal inertias of the device and sample during the measurements. Fig. 1 shows that the measured T_f values upon cooling and heating differ at rates above 10 K min^{-1} for conventional DSC and 200 K s^{-1} for FDSC. Above these scanning rates, the measurements must be corrected for thermal lag, for example by using the Tian equation:[20]

$$\Phi = \Phi_m + \tau_{in} \frac{\mathrm{d}\,\Phi_m}{\mathrm{d}t} \qquad (5)$$

where Φ and Φ_m are the corrected and measured heat flow, and τ_{in} is the time constant of the thermal inertia, which is determined from the difference of the measured fictive temperatures of the heating and cooling measurements, $\Delta T_f = T_{f,h} - T_{fc}$:

$$\tau_{in} = \frac{\Delta T_f}{2 \beta_h} \qquad (6)$$

The corrected fictive temperature is the mean value between $T_{f,h}$ and $T_{f,c}$.[17] These measurements were performed upon heating and cooling with rates between 2 and 40 K min^{-1} for DSC and 100 and 5000 K s^{-1} for FDSC. To close the gap between the two devices, additional

measurements were performed using cooling rates between 1 and 50 K min^{-1} (with DSC) and 1 and 500 K s^{-1} (with FDSC). The subsequent heating rate was 30 K min^{-1} (DSC) or 1000 K s^{-1} (FDSC). The fictive temperatures were determined from the heating measurements and corrected according to the thermal inertia. In the respective Arrhenius diagram, the logarithm of the cooling rate is plotted against the reciprocal of the limiting fictive temperature T_f (Fig. 2). The parameters of the VFTH equation are determined by best fit. The glass transition temperature is T_f at a cooling rate of 10 K/min and the fragility index was calculated from the parameters according to Eq. (4).

These values are listed in Table 1.

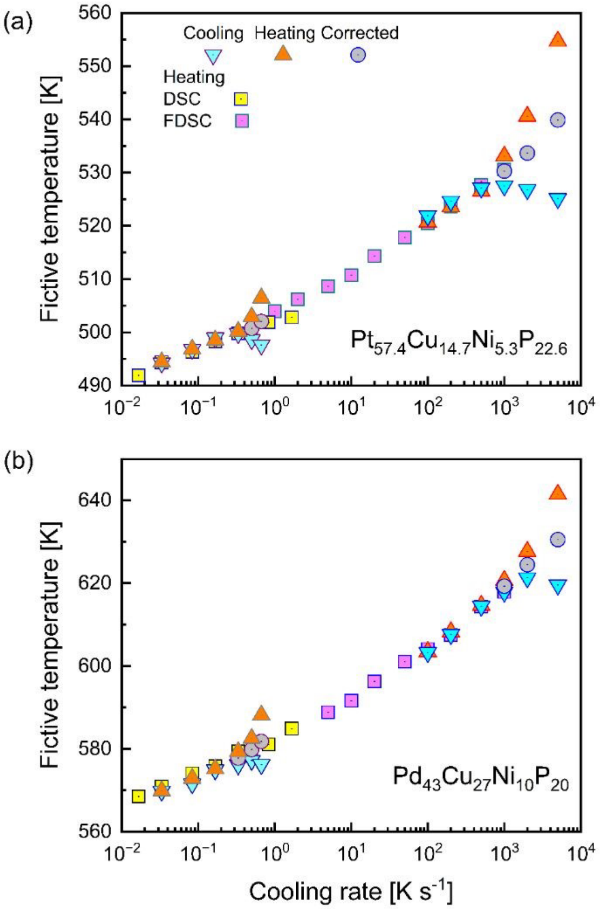

Figure 1. Limiting fictive temperature as a function of the cooling rate or the previous cooling rate for the heating measurements. The triangles are measured upon cooling (downward triangles) and subsequent heating at $\beta_h = -\beta_c$ (upward triangles), the circles are corrected data, and the squares are data measured at heating with a constant rate of 30 K min^{-1} (yellow squares) or 1000 K s^{-1} (pink squares) after cooling at various rates as indicated by the abscissa. (a) $Pt_{57.4}Cu_{14.7}Ni_{5.3}P_{22.6}$, (b) $Pd_{43}Cu_{27}Ni_{10}P_{20}$.

Table 1: Characteristic properties obtained from the Arrhenius diagrams in Figure 2.

Material	T_g [K]	A_c	B [K]	T_v [K]	m	$\xi(T_g)$ [nm]	$\mathrm{d}E_a/\mathrm{d}\xi$ [kJ mol^{-1} nm^{-1}]
$Pt_{57.4}Cu_{14.7}Ni_{5.3}P_{22.6}$	498	13.7	1310	407.9	80	2.80	376
$Pd_{43}Cu_{27}Ni_{10}P_{20}$	576	12.9	1534	463.2	69	2.65	350

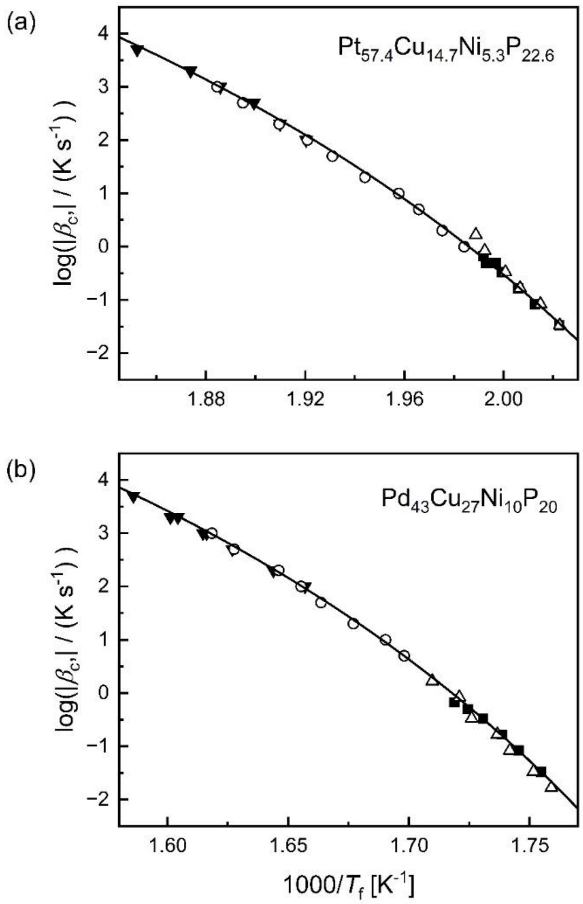

Figure 2. Arrhenius diagrams. The filled symbols are data measured at $\theta_c = -\theta_h$, while the empty symbols correspond to data measured at a constant heating rate (circles at 1000 K s^{-1} and triangles at 0.5 K s^{-1}).

(a) $Pt_{57.4}Cu_{14.7}Ni_{5.3}P_{22.6}$, (b) $Pd_{43}Cu_{27}Ni_{10}P_{20}$.

These characterizations of the macroscopic kinetics of the supercooled melts and the formation of glasses can be used to analyze the cooperative rearrangements in the melt.

The concept of characteristic subsystems with temperature fluctuations in combination with linear response theory to describe the cooperative molecular rearrangement of the α-relaxation in

supercooled melts has been developed by Donth.[21,22,23] This allows to calculate the average characteristic correlation length, ξ, of the CRR using the measured thermodynamic data:

$$\xi = \left(\frac{6}{\pi}\,\frac{k_\mathrm{B}}{\rho}\left(\frac{T_\mathrm{f}}{\delta T}\right)^2\left(\frac{1}{c_{p,\mathrm{g}}}-\frac{1}{c_{p,\mathrm{l}}}\right)\right)^{1/3} \qquad (7)$$

where T_f is the characteristic temperature of vitrification, δT is the temperature fluctuation determined from the width of the glass transition, $c_{p,\mathrm{g}}$ and $c_{p,\mathrm{l}}$ are the specific heat capacities of the glassy and liquid state, ρ is the density, and k_B is the Boltzmann constant. The validity of this approach has been recently verified by quasi-elastic neutron scattering.[24] Further validation for the applicability of this method is based on the visualization of dynamic heterogeneities in supercooled metal melts by electron correlation spectroscopy[25] and their agreement with Donth's model.[22] Furthermore, for organic materials, the correlation lengths determined by dielectric relaxation spectroscopy[26] and light scattering[27] agree well with data derived from Donth’s method.[28,29,30]

Differential scanning calorimetry (DSC), in conjunction with its fast chip-based version, allows to determine the correlation length over a wide temperature interval by studying vitrification over a ~~wide~~ large cooling-rate range.[30,31,32]
While it is likely that cooperative spatial fluctuations in highly supercooled metallic melts affect the macroscopic behavior at the glass transition,[33] such as the mechanical properties[34] and the kinetics of glass formation and crystallization, there is limited information on the correlation length, ξ, and its temperature dependence.[35] Additionally, the relationship between ξ and the alloy composition remains unclear.

In a recent work,[30] the influence of structure on the correlation length of the CRR was investigated for two polymers and the chalcogenide glass-former selenium at nearly constant macroscopic kinetics. Here, we report for the first time the measurements of $\xi(T)$ on two metallic glass-formers, which have distinct macroscopic kinetics. ~~– namely $Pt_{57.4}Cu_{14.7}Ni_{5.3}P_{22.6}$ and $Pd_{43}Cu_{27}Ni_{10}P_{20}$.~~

The ~~next step is the determination of the~~ average temperature fluctuation in the CRR. ~~This property~~ can be derived from the width of the relaxation transition ΔT_ω, which was measured using temperaturemodulated calorimetry.[31] The ΔT_ω were obtained from stochastic modulation (TOPEM) at 10 mHz in DSC and the temperature-modulated FDSC curves at 5 Hz, and correspond to the inflection temperature of the complex heat capacity curve, $|c_p^*|(T)$.[36] The width of the vitrification process during cooling, ΔT_β, is larger than ΔT_ω. This effect is described by the vitrification function $\kappa = \Delta T_\beta / \Delta T_\omega$,[17] where κ is a constant in the validity range of the VFTH equation.[17,30] For the investigated materials, κ was determined as 2.2 for $Pt_{57.4}Cu_{14.7}Ni_{5.3}P_{22.6}$ and 2.3 for $Pd_{43}Cu_{27}Ni_{10}P_{20}$. Typical cooling cures for $Pd_{43}Cu_{27}Ni_{10}P_{20}$ and the related evaluation of ΔT_β are shown in Fig. 3a.

For slowly crystallizing materials, the temperature dependence of the temperature fluctuation δT is determined directly from ΔT_ω.[31] Due to the higher crystallization tendency of ~~the~~ metals, a larger temperature range can be achieved by scanning measurements. Therefore, the temperature fluctuation δT was calculated from the vitrification width ΔT_β of the (corrected) heat flow curves by:

$$\delta T = \frac{\Delta T_\beta}{2\kappa} \qquad (8)$$

Figure 3b reveals that the temperature fluctuation increases with increasing temperature.

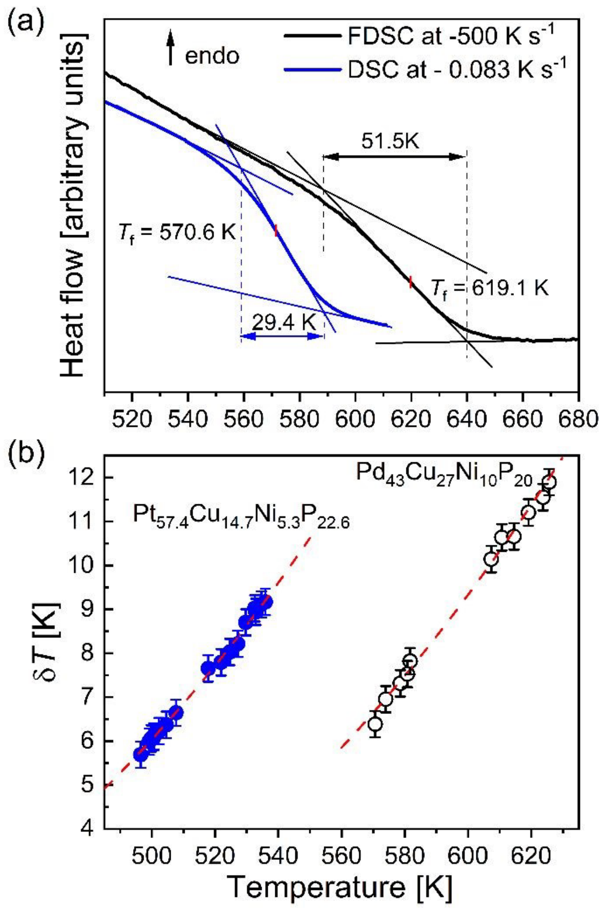

Figure 3. (a) Vitrification curves of $Pd_{43}Cu_{27}Ni_{10}P_{20}$ measured during cooling at -5 K min^{-1} (0.083 K s^{-1}) and -500 K s^{-1}, ~~with~~ and evaluation of the limiting fictive temperature and ~~the~~ width ΔT_β. (b) Average temperature fluctuation as a function of temperature. The dashed curves are guides for the eyes.

The specific heat capacities below and above the glass transition, $c_{p,g}$ and $c_{p,l}$, were measured using the TOPEM procedure. For the temperatures with known δT (see Fig. 3), the correlation length of the CRR was determined using Eq. (7). The results are plotted in Fig. 4a, and it can be seen that ξ increases with decreasing temperature. At the glass transition temperature, ξ is just below 3 nm for both glass formers. The differences between the two materials of about 0.1 nm correspond to the order of

experimental uncertainty. The correlation length is measured in a temperature interval of approximately $T_g \leq T \leq T_g$+50 K. In this range, ξ changes by ca. 50%, whereas the cooling rate and consequently the relaxation time (Eq. (2)) changes by about five orders of magnitude, as illustrated in Fig. 2. These two properties are thus not directly correlated, but it can be assumed that the change in size of the CRR is associated with the non-Arrhenius behavior of the dynamics in the supercooled melts. This can be expressed by an apparent activation energy for the relaxation process, E_a:

$$E_a(T) = -R \frac{\mathrm{d} \ln |\beta_c|}{\mathrm{d}\, (1/T_g)} = \ln 10\, R \frac{B T^2}{(T - T_V)^2} \quad (9)$$

where R is the gas constant. Thus, the apparent activation energy in glass-forming melts increases with decreasing temperature.

The comparison between E_a and ξ in Fig.4b indicates a linear relation, and thus suggests that the curvature in the Arrhenius diagram results from an increase in the size of the CRR. The slope $\frac{\mathrm{d} E_a}{\mathrm{d} \xi}$ (listed in Table 1) is then a characteristic value that describes the increase in activation energy due to the increase in correlation length of the CRR. This value is expected to correlate with the fragility index m. However, the difference between the two slopes in Fig. 4b is insignificant for the two alloys investigated. The correlation between the increase of E_a and the growth of dynamic heterogeneities with temperature decrease as well as the insensitivity of the material on the size of dynamic heterogeneities, was also observed in molecular dynamics simulations.[37]

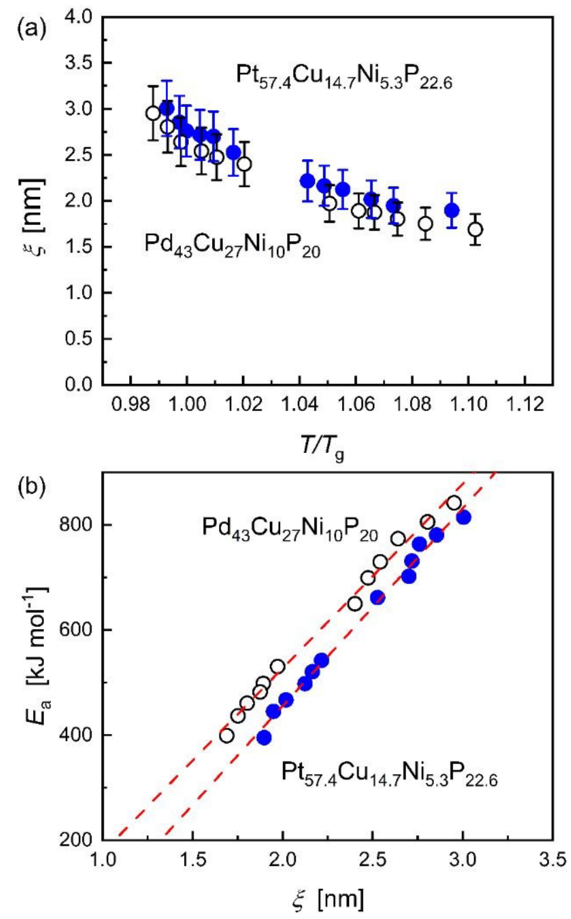

Figure 4. (a) Correlation length of the CRR as a function of reduced temperature T/T_g. (b) Apparent activation energy of the α-relaxation as a function of correlation length. The dashed lines are linear fits. The full symbols correspond to $Pt_{57.4}Cu_{14.7}Ni_{5.3}P_{22.6}$ and the open symbols to $Pd_{43}Cu_{27}Ni_{10}P_{20}$ ~~glass-forming alloys~~.

In conclusion, the measured macroscopic kinetics of the metallic melts and their vitrification a consistent with those of low-molecular organic materials and polymers. Therefore, the atomic (or molecular) origins for their macroscopic behavior are most likely comparable for different classes of materials. This is supported by the observation of dynamic heterogeneities in supercooled metallic

melts close to vitrification.[25] bBy combining conventional DSC and FDSC, and after correcting the influence of thermal inertia, the activation curves of the vitrification process and the correlation length of the cooperative rearrangement regions (CRR) has been determined in a temperature range slightly below T_g and about 50 K above T_g for two glass-forming alloys. The temperature range was wide enough to determine the macroscopic kinetic parameters (i.e., the Vogel-Fulcher-Tammann-Hesse (VFTH) parameters A, B and T_v) and the fragility parameter m. The correlation length ξ of the CRR increases from about 1.8 nm at $T/T_g \approx 1.1$ to ca. 3 nm at T_g. Surprisingly, no significant difference between the two materials has been found for the absolute value of ξ and its dependency as a function of reduced temperature T/T_g, despite the fact that the kinetic parameters differ largely. This leads us to conclude that the compositional differences between the two glass-forming alloys are relevant for the macroscopic kinetic parameters but less important for the dimensions of the temporary heterogeneities associated with CRR. The increase in ξ with decreasing temperature appears to relate with the change in apparent activation energy of the cooperative atomic movements. From this we can draw This leads to three conclusions regarding the composition, the correlation length ξ, and the macroscopic kinetics:

(1) The composition and the related atomic interaction strongly influence the VFTH parameters and the glass transition temperature.

(2) The size of the dynamic fluctuations, relevant for the cooperative rearrangements near the glass transition, and its dependency on the reduced temperature T/T_g are not critically influenced by the composition.

(3) The increasing size of the spatial-temporal fluctuating domains with decreasing temperature correlates with the apparent activation energy of the relaxation process.

This means that the increase in ξ with decreasing temperature is the reason for the nonArrhenius behavior of the α-relaxation.

EXPERIMENTAL METHODS

Cylindrical ingots of master alloys with a nominal composition of $Pd_{43}Cu_{27}Ni_{10}P_{20}$ and $Pt_{57.4}Cu_{14.7}Ni_{5.3}P_{22.6}$ (at.%)[38] were acquired from the PX Group, Switzerland. They were cut into small pieces, weighing around 4 grams, which were used to prepare amorphous ribbons via the melt spinning technique: the master alloy pieces were inductively melted in quartz crucibles, and then ejected through a nozzle of 1 mm diameter. The casting parameters were kept identical for both alloys. In detail, the nozzle-to-wheel distance was set to 0.3 mm, the ejection overpressure was 300 mbar and the Cu-wheel speed was 20 m/s. Although the melt spinning device is equipped with a two-color pyrometer, the actual ejection temperature could not be measured due to the low melting points of both alloys. The chamber was initially evacuated down to a pressure of 4×10^{-5} mbar, and for the melting and melt spinning it was filled with Ar 6.0 purity (99.9999 %) up to a pressure of 500 mbar. The melt-spun ribbons were 1 mm wide and around 20 µm thick.

The densities of the two alloys were taken from literature, and are ρ = 15.020 g cm^{-3} for $Pt_{57.4}Cu_{14.7}Ni_{5.3}P_{22.6}$ [10] and ρ = 9.425 g cm_{-3} for $Pd_{43}Cu_{27}Ni_{10}P_{20}$.[11]

Conventional DSC measurements were performed using a DSC823^{e} Mettler-Toledo device equipped with a sample robot and cryostat. The ribbons were cut into small sections and placed in an oxidized Al crucible. The cell gas was argon and the protective gas was nitrogen. The typical sample size was 60 mg.

The temperature range for the Pt-based alloy was between 170 and 275 °C, and for the Pd-based alloy it was between 220 and 350 °C.

Temperature-modulated DSC measurements were performed using the stochastic modulation mode (TOPEM)[39] of the DSC823^{e} device. The measurements were carried out during cooling with a rate of 1 K min^{-1}. The measurement parameters are a temperature amplitude of 1 K and switching times of 30 and 31 s. The evaluation parameters relate to a calculation window of 200 s, a sample response parameter of 20, and an instrumental response parameter of 45. These measurements were performed to determine the heat capacity, and the heat capacity relaxation at 10 mHz.

A Flash DSC 2+ (Mettler-Toledo) with an IntraCooler TC100 (Huber) and a UFH 1 sensor were used for fast DSC (FDSC) measurements. The cell gas was Ar. A thin oil film was prepared on the sample side before inserting the sample with a typical weight of 1 µg. The temperature ranges were 25 °C ≤ T ≤ 320 °C and 25 °C ≤ T ≤ 440°C, respectively. Temperature-modulated measurements at 5 Hz were carried out with a saw-tooth modulation during cooling from the supercooled melt with an amplitude of 1 K and an underlying cooling rate of 2 K/s (further details in Ref. 40).

AUTHOR INFORMATION

Corresponding Author

Jürgen E.K. Schawe – *Laboratory of Metal Physics and Technology, Department of Materials, ETH Zurich, 8093 Zurich, Switzerland;*

Email: juergen.schawe@mat.ethz.ch

Authors

Min Kyung Kwak - *Laboratory of Metal Physics and Technology, Department of Materials, ETH Zurich, 8093 Zurich, Switzerland; Department of Materials Science and Engineering, Research Institute of Advanced Materials & Institute of Engineering Research, Seoul National University, Seoul 08826, Republic of Korea*

Mihai Stoica - *Laboratory of Metal Physics and Technology, Department of Materials, ETH Zurich, 8093 Zurich, Switzerland*

Eun Soo Park - *Department of Materials Science and Engineering, Research Institute of Advanced Materials & Institute of Engineering Research, Seoul National University, Seoul 08826, Republic of Korea*

Jörg F. Löffler - *Laboratory of Metal Physics and Technology, Department of Materials, ETH Zurich, 8093 Zurich, Switzerland*

AUTHOR CONTRIBUTIONS

J.E.K.S.: Conceptualization, Methodology, Investigation, Writing original draft. M.K.K.: Formal analysis, Investigation, Writing review and editing. M.S.: Investigation, Writing original draft. E.S.P.: Funding acquisition, Writing review and editing. J.F.L.: Conceptualization, Resources, Project administration, Writing review and editing.

ACKNOWLEDGEMENTS

The authors gratefully acknowledge the support of the ETH+ initiative within the framework of

SynMatLab (Laboratory for Multiscale Materials Synthesis and Hands-On Education). ESP and MKK were supported by the Creative Materials Discovery Program through the National Research Foundation of Korea (NRF) funded by the Ministry of Science and ICT (No. NRF-2019M3D1A1079215).

REFERENCES

(1) Löffler, J. F.; Schroers, J.; Johnson, W.L. Time-temperature-transformation diagram and microstructures of bulk glass forming Pd40Cu30Ni10P20. *Appl. Phys. Lett.* **2000**, *77*, 681–683.

(2) Shen, Y.; Perepezko, J.H. Al-based amorphous alloys: glass-forming ability, crys- tallization behavior and effects of minor alloying additions. *J. Alloys Comp.* **2017**, *707*, 3–11.

(3) R. Busch, The thermodynamical properties of bulk metallic glass-forming liquids, JOM 52 (2000) 39–42.

(4) Wang, X.; Xu, W.-S.; Zhang, H.; Douglas J.F. Universal Nature of Dynamic Heterogeneity in GlassForming Liquids: A Comparative Study of Metallic and Polymeric Glass-Forming Liquids. *J. Chem.*
Phys. **2019**, *151*, 184503.

(5) Debenedetti, P.G.; Stillinger, F.H. Supercooled Liquids and the Glass Transition. *Nature* **2001**, *410*, 259−267.

(6) Adam, G.; Gibbs, H.J. On the Temperature Dependence of Cooperative Relaxation Properties in Glass-Forming Liquids. *J. Chem.Phys.* **1965**, *43*, 139−146.

(7) Berthier, L. Self-Induced Heterogeneity in Deeply Supercooled Liquids. *Phys. Rev. Lett.* **2021**, *127*,

088002.

(8) Starr, W.F.; Douglas, J.F.; Sastry, S. The relationship of dynamical heterogeneity to the Adam-Gibbs and random first-order theories of glass formation, *J. Chem. Phys.* **2013**, *138*, 12A541.

(9) Ozawa, M.; Scalliet, C.; Ninarello, A.; Berthier, L. Does the Adam-Gibbs relation holds in simulated supercooled liquids? *J. Chem. Phys.* **2019**, *151*, 084504.

(10) Gopinadhan, M.; Shao, Z.;Liu, Y.; Mukherjee, M.; Sekol, R. C.; Kumar, G.; Taylor, A. D.; Schroers, J.; Osuji, C.O. Finite size effects in the crystallization of a bulk metallic glass. *Appl. Phys. Lett*. **2013**, *103*, 111912.

(11) Lu, I.-R.; Görler, G. P.; Fecht, H. J.; Willnecker, R. Investigation of specific volume of glass-forming Pd-Cu-Ni-P alloy in the liquid, vitreous and crystalline state. *J. Non-Cryst. Solids* **2002**, *312–314*, 547–551.

(12) Vogel, H. Temperaturabhängigkeitsgesetz der Viskosität von Flüssigkeiten. *Phys. Z.* **1921**, *22*, 645–646.

(13) Fulcher, G. S. Analysis of recent measurements of the viscosity of glasses. J. *Am. Ceram. Soc.* **1925**, *8*, 339–355.

(14) Tammann, G.; Hesse, W. Die Abhängigkeit der Viskosität von der Temperatur von unterkühlten Flüssigkeiten. *Z. Anorg. Allg. Chem*. **1926**, *156*, 245–257.

(15) Moynihan, C. T.; Easteal, A. J.; DeBolt, M. A.; Tucker, J. Dependence of the fictive temperature of glasses on cooling rate. *J. Am. Ceram. Soc.* **1976**, *59*, 12–16.

(16) Richardson, M. J.; Savill, N. G. Derivation of accurate glass transition temperatures by differential scanning calorimetry. *Polymer* **1975**, *16*, 753–757.

(17) Schawe, J. E. K. Vitrification in a wide cooling rate range: The relations between cooling rate, relaxation time, transition width, and fragility. *J. Chem. Phys*. **2014**, *141*, 184905.

(18) Angell, C. A. Spectroscopy Simulation and Scattering, and the Medium Range Order Problem in Glass. *J. Non-Cryst. Solids* **1985**, *73*, 1−17.

(19) Schawe, J. E. K. An analysis of the meta stable structure of poly(ethylene terephthalate) by conventional DSC. *Thermochim. Acta* **2007**, *461*, 145-152.

(20) Vyazovkin, S.; Sbirrazzuoli, N. Nonisothermal crystallization kinetics by DSC: Practical overview. *Processes* **2023**, *11*, 1438.

(21) Donth, E. The size of cooperatively rearranging regions at the glass transition. *J. Non-Cryst. Solids* **1982**, *53*, 325-330.

(22) Donth, E. Phenomenological treatment of dynamic glass transition heterogeneity. *Acta Polym*. **1999**, *50*, 240 – 251.

(23) Donth, E. The glass transition, Springer-Verlag, Berlin, Heidelberg **2001**.

(24) Chua, Y. Z.; Zorn, R.; Schmelzer, J. W. P.; Schick, C.; Holderer, O.; Zamponi, M. Determination of Cooperativity Length in a Glass-Forming Polymer. *ACS Phys. Chem. Au* **2023**, *3*, 172−180.

(25) Zhang, P.; Maldonis, J. J.; Liu, Z.; Schroers, J.; Voyles, P. M. Spatially heterogeneous dynamics in a metallic glass forming liquid imaged by electron correlation microscopy. *Nat. Comm*. **2018**, *9*, 1129.

(26) Cangialosi, D., Alegría, A.; Colmenero, J. Dielectric relaxation of polychlorinated biphenyl/toluenemixtures: Component dynamics. *J. Chem. Phys.* **2008**, *128*, 224508.

(27) Hong, L., Novikov, V.N.; Sokolov, A.P. Dynamic heterogeneities, boson peak, and activation volume in glass-forming liquids. *Phys. Rev E* **2011**, *83*, 061508.

(28) Hempel, E.; Hempel, G.; Hensel, A.; Schick, C.; Donth, E. Characteristic length of dynamic glass transition near Tg for a wide assortment of glass-forming substances. *J. Phys. Chem. B* **2000**, *104*, 2460-2466.

(29) Rijal, B., Delbreilh, L.; Saiter, A. Dynamic Heterogeneity and Cooperative Length Scale at Dynamic Glass Transition in Glass Forming Liquids. *Macromolecules* **2015**, *48*, 8219–8231.

(30) Hallavant, K.; Mejres, M.; Schawe, J. E. K.; Esposito, A.; Saiter-Fourcin, A. Influence of chemical composition and structure on the cooperative fluctuation in supercooled glass-forming liquids. *J. Phys. Chem. Lett.* **2024**, *15*, 4508–4514.

(31) Chua, Y. Z.; Schulz, G.; Shoifet, E.; Huth, H.; Zorn, R.; Schmelzer, J. W. P.; Schick, C. Glass transition cooperativity from broad band heat capacity spectroscopy. *Colloid Polym. Sci.* **2014**, *292*, 1893–1904.

(32) Schawe, J. E. K. Measurement of the thermal glass transition of polystyrene in a cooling rate range of more than six decades. *Thermochim. Acta* **2015**, *603*, 128-134.

(33) Wei, S.; Everson, Z.; Galano, I.; Busch, R. The impact of fragility on the calorimetric glass transition
in bulk metallic glass. *Intermetallics* **2014**, 55, 138-144.

glasses from shear transformation zone dilatation.

(34) Jiang, M .Q.; Wilde, G.; Jiang, F.; Dai, L. H. Understanding ductile-to-brittle transition of metallic

Theoretical and Applied Mechanics Letters,

2015, *5*, 200-204.

(35) Wang, T.; Li, J. B.; Yang, J. Y.; Rao, G. H. A comparative study on characteristic relaxation in
$Ce_{65}Al_{10}Cu_{20}Co_5$ and $Zr_{46.75}Ti_{8.25}Cu_{7.5}Ni_{10}Be_{27.5}$ bulk metallic glasses and supercooled liquids.
Intermetallics **2011**, *19*, 81-85.

(36) Schawe, J. E. K. Principles for the interpretation of modulated temperature DSC measurements. Part 1. Glass transition. *Thermochim. Acta* **1995**, *261*, 183-194.

(37) Zhang, H.; Wang, X.; Yu, H.-B.; Douglas, J. F. Dynamic heterogeneity, cooperative motion, and Johari-Goldstein β-relaxation in a metallic glass-forming material exhibiting a fragile-to-strong transition, *Eur. Phys. J. E* **2021**, *44*, 56.

(38) Haag, F.; Sauget, R.; Kurtuldu, G.; Prades-Rödel, S.; Schawe, J. E. K.; Blatter, A.; Löffler, J. F. Assessing continuous casting of precious bulk metallic glasses. *J. Non-Cryst. Solids* **2019**, *521*, 119120.

(39) Schawe, J. E. K.; Hütter, T.; Heitz, C.; Alig, I.; Lellinger, D. Stochastic temperature modulation: A new technique in temperature-modulated DSC. *Thermochim. Acta* **2006**, *446*, 147-155.

(40) Lindemann, N.; Schawe, J. E. K.; Lacayo-Pineda, J. Kinetics of the glass transition of silica-filled styrene-butadiene-rubber: The effect of resins. *Polymers* **2022**, *14*, 2626.

jz-2024-03275v.R2

Name: Peer Review Information for "The cooperativity of atomic fluctuations in highly supercooled glass-forming metallic melts"

Second Round of Reviewer Comments

Reviewer: 2

Comments to the Author

--First of all, let me point out that although the Reviewer #1 gave you very positive opinion, it has zero scientific inputs. You may dislike some of my comments. Me, as the reviewer #2, was trying my best to improve the quality of your work. Actually, the marks I gave was not bad. I realized from your response letter that the authors may be upset about some of my questions, but please have some respects, considering how much effort I have put on reviewing your article! At the end, you could always ask the editor to seek opinions from another reviewer if you think I am not qualified.

--Comments on the revised manuscript:

--1. Visualization of dynamic heterogeneities in supercooled melts

--I am glad that you included many relevant papers into this revised version, especially this one: P. Zhang, et al. Nature Communications 9 (2018) 1129. I have no doubts that dynamic heterogeneities or CRR could exist in glasses, and the average size shifts with temperature, but it doesn't mean that you have measured the right size of CRR from your calorimetry curves. Connecting delta_T (in unit of Kelvin) to epsilon (in unit of nm) is not straight-forwards, unless you could provide spatial evidence using approaches like correlation spectroscopy on your samples. Zhang worked on metallic glasses but didn't measure calorimetry. Even if we neglect the compositional difference between yours and theirs, they used nanowires for electron correlation spectroscopy, but you used micrometer size particles in fast calorimetry. Would sample size matter for epsilon? This is unanwsered! Furthermore, Zorn studied polymers, and they assumed a spherical shape for CRR to calculate epsilon (Eq. 7). Is the shape of CRR for metallic glasses testified? Moreover, why is density in Eq. 7 a constant for calculating epsilon? It may vary upon vitrification, too.

--My point is that you have only measured delta_T, which is a value that can be modified depending on what percentage you chose for glass-transition intensity of specific heat. An absolute value of epsilon can not be asserted by Eq. 7, unless you could confirm the linkage between size the temperature by experiments. So far, you have only shown the trend of delta_T(Tf) is consistent to an increasing size of CRR with the decreasing temperature, but nothing more.

P.S. Is Ref. 13 or 13a the correct number you quoted?

--2. Interpretations of delta_T or vitrification width in calorimetry curves.

--I asked you to discuss other possibilities on how to interpret delta_T. I agree with you that no serious discussion has been made yet on interpreting delta_T for relaxation time. But, so did not for CRR size. That is why we need to make a comparison before rushing to your conclusion.

--3. About Ea or the apparent activation energy.

--The reason I dislike to use a temperature-dependent Ea for alpha relaxation is because it lacks a fundamental physical picture. For calculating Ea, one needs to assume an Arrenhius relationship first, which is fine for beta relaxation. For alpha relaxation, we both knew metallic glasses follow the VFT relationship. So, if it is already non-Arrenhius, why bother to make a statement of Ea under the assumption of Arrenhius? Also, a single value of Ea helps to distinguish beta relaxation, but a range of Ea does not help for the definition of alpha relaxation. It doesn't make sense to me to define alpha relaxation under the framework of Arrenhius. （It is OK if you disagree with me, but please mention why Ea is called an apparent activation energy in text.)

--The point you made is that the slope of cooling-rate dependent Tf changes (obeying VFT) has some connections to epsilon. This is OK as the changing slope is a signature of fragile glasses. Many theories are proposed to explain fragility, but the central theme is about heterogeneities in the supercooled liquid. However, I doubt that Ea and epsilon will be a linear curve as showed by the dashed lines in Fig. 4b. Epsilon is two thirds in power to Tf (Eq. 7) and Ea is a square of Tf (Eq. 9), there is no reason to believe that they are linear. The linear fits in Fig. 4b may just reflect a limited range of your data.

--P.S. In Fig. 4a and Fig. 3b, the X-axes should be Tf?

--In conclusion, the authors should make a clear separation between experimental data and their interpretation. Try not to make a decisive argument unless solid evidence is available.

Author's Response to Peer Review Comments:

Response to the Editor and the Reviewers to the First Revision

We would like to thank again the reviewer for his/her work and appreciate the reviewer's suggestions.

Changes have been made as explained in the following and as identified in the appended highlighted version of the revised manuscript.

Response to Reviewers' comments

Reviewer #2

(1) Recommendation: This paper may be publishable, but major revision is needed; I would like to be invited to review any future revision.

.

Authors:

Thank you for your work and your comments.

(2) First of all, let me point out that although the Reviewer #1 gave you very positive opinion, it has zero scientific inputs.

Authors:

We assume that the editor selected reputable scientists for the reviewing process. We thus appreciate the positive comments by Reviewer #1, who is expected to be an experienced scientist working in the broad field of glasses.

(3) You may dislike some of my comments. Me, as the reviewer #2, was trying my best to improve the quality of your work. Actually, the marks I gave was not bad. I realized from your response letter that the authors may be upset about some of my questions, but please have some respects, considering how much effort I have put on reviewing your article! At the end, you could always ask the editor to seek opinions from another reviewer if you think I am not qualified.

Authors:

We appreciate the work of all the reviewers. Unfortunately, Reviewer #2 seems to have misinterpreted our comments as personal criticism. In fact, we simply disagree with the Reviewer #2 on some comments and have therefore tried to express ourselves as precisely as possible.

In our opinion this has nothing to do with like or dislike. This is just a part of scientific discussions.

(4) I am glad that you included many relevant papers into this revised version, especially this one: P. Zhang, et al. Nature Communications 9 (2018) 1129. I have no doubts that dynamic heterogeneities or CRR could exist in glasses, and the average size shifts with temperature, but it doesn't mean that you have measured the right size of CRR from your calorimetry curves.

Authors:

This is an important misinterpretation of our work. As already mentioned in our last answer, the behavior and properties of a glass are not the subject of our work. The topic is the properties of supercooled glass-forming melts. Unlike the reviewer, we have doubts about the existence of CRR in glasses. Moreover, we do not use the magnitude of heterogeneities as measured in the

mentioned work as quantitative evidence. We interpret this interesting work as the first experimental visualization of dynamic heterogeneities. Quantitatively, there are some doubts about the data, as e.g. the non-planar sample might influence the spatial and temporal resolution. We have clarified this point in the manuscript.

Page 8: The dynamic heterogeneities slightly above the glass transition temperature were visualized by electron correlation spectroscopy using the example of metallic glass nano-wires.[30], and the result agrees with Donth's model for CRR.[26]

(5) Connecting delta_T (in unit of Kelvin) to epsilon (in unit of nm) is not straight-forwards, unless you could provide spatial evidence using approaches like correlation spectroscopy on your samples. Zhang worked on metallic glasses but didn’t measure calorimetry.

Authors:

We think this is a typographical error. We use xi as the symbol for the correlation length and not epsilon.
As we mentioned in our first reply, we waited for an experimental confirmation of the thermodynamic basis in literature before applying the Donth approach. This has meanwhile been done in literature by the recent comparison with neutron scattering and calorimetry using the example of polymers (Ref. 29). For thermodynamic models without structural assumptions, the material used is not decisive. For the derivation of equation (7) (relationship between xi and delta_T), please refer to references 25-27.
There is a fundamental difference between the visualized heterogeneities in Ref. 30 and the CRR. The CRR are thermodynamically defined as the smallest representative thermodynamic subsystem. This means that all fluctuations in the CRR (on average) reflect the macroscopic sample in its dynamics. The CRR must also include fast and slow relaxations and are larger than the regions of increased mobility. Von Laue already mentioned the relationship between temperature fluctuations and the size of a subsystem already in a paper published in 1917 (Ref. 6).

A possible thermodynamic model was discussed in Ref. 26. In this sense, the fast domains with a size of about 1 nm fit very well into the model. However, for a qualitative analysis of the heterogeneities in the melt, the method used in Ref. 27 needs to be improved in terms of temporal and spatial resolution.

The classical Adam-Gibbs theory is also based on von Laue’s thermodynamics. Tatsumi et al. (Ref. 35) determined the number of particles in the CRR by precise measurements of the heat capacity in the melt. This result can also be easily converted into a spatial length. We have presented the relationship between delta_T and xi in more detail and have added further references.

Page 8: This thermodynamic approach is based on von Laue’s thermodynamics of temperature fluctuations,[6] which was further developed by Landau and Lifshitz.[28] The validity of this approach has been recently verified by quasielastic neutron scattering for polymeric materials.[29]

Page 8: Furthermore, for organic materials, the correlation lengths determined by dielectric relaxation spectroscopy,[31,32] NMR[33], light scattering[34] and direct measurements of the configurational entropy at low temperature[35] agree well with data derived from Donth's method.[36,37,24]

Although these methods have been applied to non-metallic glass-forming liquids, Donth's approach is based on a validated thermodynamic concept without assumptions about molecular or atomic structure and interaction. We can therefore also safely apply it to atomic systems.

Additional references:

(6) von Laue, M. Temperatur und Dichteschwankungen, *Physik. Z.* **1917**, *18*, 542-544.

(28) Landau, L.D, Lifshitz, E.M, Statistical physics part 1, third edition, Pergamon Press, **1980.**

(32) Berthier, L.; Biroli, G.; Bouchaud, J.-P.; Cipelletti, L.; El Masri, D.; L'Hote, D.; Ladieu, F.; Pierno, M. Direct experimental evidence of a growing length scale accompanying the glass transition. *Science* **2005**, 310, 1797-1800.

(33) Qiu, X. H.; Ediger, M. D. Length scale of dynamic heterogeneity in supercooled D-sorbitol: comparison to model predictions. *J. Phys. Chem. B* **2003**, *107*, 459-464.

(35) Tatsumi, S.; Aso, S.; Yamamuro, O. Thermodynamic study of simple molecular glasses: Universal features in their heat capacity and the size of the cooperatively rearranging regions. *Phys. Rev. Lett.* **2012**, *109*, 045701.

(6) Even if we neglect the compositional difference between yours and theirs, they used nanowires for electron correlation spectroscopy, but you used micrometer size particles in fast calorimetry.

Authors:

This problem often occurs in all types of investigations via transmission electron microscopy (TEM), where micro- and nanoscale samples are used. There are always doubts about the direct upscaling of the results and the representativeness of the micrographs for macroscopic samples. Another major potential problem is the possible influence of the preparation technique on the specimen. Since this dilemma is not the subject of the paper, we have not discussed this in detail. Here we only refer to Ref. 30 to illustrate the existence of dynamic heterogeneities.

(7) Would sample size matter for epsilon? This is unanwsered!

Authors:

We are sorry that we overlooked this point in our last answer.
There are many studies on the size dependence of vitrification with controversial experimental results. But more and more the results tend towards the conclusion that there might be a size dependence starting from about 10 nm or below. We will not discuss this in the manuscript as this is a different topic and the discussion is still ongoing.
The samples we used behave macroscopically and are chosen such that there is neither a size dependence nor an influence of static temperature gradients or mechanical stress (see e.g. https://doi.org/10.1007/978-3-319-31329-0_1).

(8) Furthermore, Zorn studied polymers, and they assumed a spherical shape for CRR to calculate epsilon (Eq. 7). Is the shape of CRR for metallic glasses testified?

Authors:

In Zorn's work, a polymer melt was investigated by comparing calorimetry and neutron scattering. It seems that such measurements are complicated for highly supercooled metal melts due to their lower stability to structural changes such as nucleation and crystallization. The objectives of our work were to prove the validity of a thermodynamic approach, namely the von Laue thermodynamics of temperature and density fluctuations. This test came out positive. A general thermodynamic method that does not use specific material models is generally valid for all thermodynamic systems, regardless of the type of particles involved (atoms, molecules, macromolecules or colloids). Thus, from a thermodynamic point of view, there is no doubt about the existence of CRR in supercooled molten metals, but the details, e.g. the size and its temperature change, are unknown.

This was the starting point of our work. The first step was to measure the kinetics of vitrification, and the results were somewhat surprising: We could not find any significant differences between organic substances, polymers, melts of chalcogenide glass formers and metal melts. This finding is consistent with the results of molecular dynamics simulations (Ref. 45).

It is possible that this point was not mentioned clearly enough in the paper. We thus modified the text accordingly.

Page 8: Although these methods have been applied to non-metallic glass-forming liquids, Donth's approach is based on a validated thermodynamic concept without assumptions about molecular or atomic structure and interaction. We can therefore also safely apply it to atomic systems. (see response to point (5))

Page 11: …, and the insensitivity of the material to the size of dynamic heterogeneities were also observed in molecular dynamics simulations by the comparison of polymers and metals.[45]

(9) Moreover, why is density in Eq. 7 a constant for calculating epsilon? It may vary upon vitrification, too.

Authors:

The density is important for the absolute value of the correlation length, but not for the temperature dependence, as the term (Tf/delta_T)^2 changes by a factor of 3 in the investigated temperature range, but the density change is well below 10%. This means that the density change influences the reported data by less than 3%. With an uncertainty of around 15%, this is negligible. A comment is added.

Page 9: The values given in literature were used for the density.[11,12] The small change in ρ with temperature was neglected as it only has a minor influence on the uncertainty of ξ.

(10)--My point is that you have only measured delta_T, which is a value that can be modified depending on what percentage you chose for glass-transition intensity of specific heat. An absolute value of epsilon can not be asserted by Eq. 7, unless you could confirm the linkage between size the temperature by experiments. So far, you have only shown the trend of delta_T(Tf) is consistent to an increasing size of CRR with the decreasing temperature, but nothing more.

Authors:

The reviewer is right. The determination of delta_T is associated with experimental uncertainties. The use of the half-life is based on a Gaussian distribution. We have applied the most robust evaluation methods and evaluated all curves according to the same standards, but ultimately the relative experimental uncertainty for the correlation length is in the order of +/- 15%, as shown in Fig. 4a. The absolute uncertainty of the correlation length can be estimated by comparing different literature data. For different authors, different materials and different techniques, this uncertainty can be estimated at 0.5 nm.
We added a comment on the experimental uncertainty.

Page 9: The relative experimental uncertainty of ξ is approximately ±15 %. When comparing different methods and different materials, the absolute error is in the order of 0.5 nm.

(11) P.S. Is Ref. 13 or 13a the correct number you quoted?

Authors:

We apologize for this misprint. The reference was P. Zhang, et al. Nature Communications 9 (2018) 1129.

(12) Interpretations of delta_T or vitrification width in calorimetry curves.
--I asked you to discuss other possibilities on how to interpret delta_T. I agree with you that no serious discussion has been made yet on interpreting delta_T for relaxation time. But, so did not for CRR size. That is why we need to make a comparison before rushing to your conclusion.

Authors:

Thank you very much. We could not understand this question in the first report. Now it is clearer for us.
A detailed discussion of the significance of delta T can be found in J. Chem. Phys. 2014, 141, 184905. It is directly related to the width of the relaxation spectrum of alpha relaxation. This is not surprising since all fluctuations associated with relaxation also correlate with temperature fluctuations. Using macroscopic kinetics, the width of the relaxation spectrum is converted into the temperature width of this relaxation using the VFTH equation. This means that the relaxation spectrum is fitted e.g. with the Havriliak-Negami function or the Kohlrausch function and the relaxation time is replaced by the VFTH equation to obtain the temperature dependence. The half-width can then be determined. This would be 2 delta_T. Looking at the vitrification, the transition becomes wider because the change in relaxation time slows down when leaving equilibrium. Phenomenologically, this is described by the constant kappa. Other methods are known, e.g. using the concept of fictitious temperature and reduced time by Tool and Narayanaswamy.

In the manuscript, we added comments on the relationship between the relaxation spectrum and the temperature fluctuation.

Page 7: A frequently discussed kinetic parameter is the width of the relaxation spectrum, which describs the distribution of relaxation times. For the α relaxation, the temperature width ΔT_ω, which is measured by modulated calorimetry at the frequency ω, is directly related to the width of the relaxation spectrum $\Delta\ln\omega$.[18] From

$$\frac{\Delta\ln\omega}{\Delta T_\omega} \approx \frac{\mathrm{d}\ln\omega}{\mathrm{d}T} \tag{7}$$

with $\omega \approx 1/\tau$ we obtain from Eq. (1) for the relation between the width of temperature dispersion measurements and the corresponding results for frequency dispersion in thermo-rheological simple liquids.

$$\Delta T_\omega \approx \Delta\ln\omega \frac{\ln 10\,(T - T_V)^2}{B} \tag{8}$$

Page 7: As expected, the temperature width increases with increasing temperature of the α relaxation.

Page 9: …where the constant κ considers the broadening of the transformation interval during vitrification due to the slowing down of the change of the relaxation time.[18]

(13) About Ea or the apparent activation energy.

--The reason I dislike to use a temperature-dependent Ea for alpha relaxation is because it lacks a fundamental physical picture. For calculating Ea, one needs to assume an Arrenhius relationship first, which is fine for beta relaxation. For alpha relaxation, we both knew metallic glasses follow the VFT relationship. So, if it is already non-Arrenhius, why bother to make a statement of Ea under the assumption of Arrenhius? Also, a single value of Ea helps to distinguish beta relaxation, but a range of Ea does not help for the definition of alpha relaxation. It doesn't make sense to me to define alpha relaxation under the framework of Arrenhius.

Authors:

The reviewer seems to define the activation energy only in the context of the Arrhenius equation. In fact, the activation energy is defined as the activation barrier for the transformation of a state A into a state B. The ratio between Ea and RT is the thermodynamic probability for the transformation. Arrhenius derived his equation for the case where the temperature dependence of the activation barrier is negligible. This is found experimentally, for example, in the case of short-range Johari-Goldstein beta relaxation.

In long-range cooperative relaxation, the temperature dependence of the free volume has a significant impact on the activation barrier. This is the reason for the temperature dependence of the energy landscape and hence of Ea.

It seems unlikely that long-range fluctuations are not thermally activated, unlike short-range fluctuations. The alpha process follows the comparable behavior as the viscosity and the diffusion constant. The classical Turnbull-Fisher theory of nucleation describes the probability of nucleation as the product of the thermodynamic probability of nucleation and the probability of particle transport, the latter being given by the activation barrier of long-range transport, which is usually described by the activation energy of the alpha relaxation

To convince the reviewer that the thermal activation of the alpha process does not correspond only to the authors' individual understanding of thermodynamic fluctuations, we added exemplarily references from a large reference list to this topic.

Page 10: The concept of thermally activated cooperative rearrangements is widely accepted in literature.[42,43,44]

Additional references

(42) Annamareddy A.; Voyles, P. M.; Perepezko, J.; Morgan, D. Mechanisms of bulk and surface diffusion in metallic glasses determined from molecular dynamics simulations, *Acta Mater*. **2021**, *209*, 116794

(43) Liu, C.; Pineda, E.; Crespo, D. Mechanical relaxation of metallic glasses: An overview of experimental data and theoretical models. *Metals* **2015**, *5*, 1073-1111.

(44) J.M. Pelletier, J. M.; Cardinal, S.; Qiao, J. C.; Eisenbart, M.; Klotz, U. E. Main and secondary relaxations in an Au-based bulk metallic glass investigated by mechanical spectroscopy. *J. Alloys Compd*. **2016**, *684*, 530-536

(14) （It is OK if you disagree with me, but please mention why Ea is called an apparent activation energy in text.)

Authors:

The term "apparent activation energy" is a common expression in the kinetic literature and in physical chemistry. The term "apparent" describes the fact that Ea is a macroscopic measurement result, but the atomic processes can be more complex. As a simple example, we can again use the Turbull-Fisher theory of nucleation. In this case, the apparent activation energy is the sum of the thermodynamic and transport barriers, both of which have different temperature dependences.

(15)--The point you made is that the slope of cooling-rate dependent Tf changes (obeying VFT) has some connections to epsilon. This is OK as the changing slope is a signature of fragile glasses. Many theories are proposed to explain fragility, but the central theme is about heterogeneities in the supercooled liquid. However, I doubt that Ea and epsilon will be a linear curve as showed by the dashed lines in Fig. 4b. Epsilon is two thirds in power to Tf (Eq. 7) and Ea is a square of Tf (Eq. 9), there is no reason to believe that they are linear. The linear fits in Fig. 4b may just reflect a limited range of your data.

Authors:

We thank the reviewer for this comment. The reviewer is right, the only reason for the linear approach is the small interval of xi in which we can measure. To see the full temperature dependence of xi, one must measure up to temperatures close to the split point between the Johari-Goldstein relaxation and the alpha relaxation, which is expected in the region of the melting temperature. Experimentally, this cannot be performed.
To a good approximation, xi is proportional to Ea^(2/3). This is discussed now in the Supplementary Information.

Page 10: A more extensive discussion of the relation between E_a and ξ is given in the Supplementary Information.

(16)--P.S. In Fig. 4a and Fig. 3b, the X-axes should be Tf?

Authors:

Thank you for the suggestion. The limiting fictive temperature is characteristic of the vitrified state. We determine parameters of the melt for which the fictive temperature is equal to the temperature. The correction used is based on the shape parameter kappa, Eq. (10).

(17)--In conclusion, the authors should make a clear separation between experimental data and their interpretation.

Authors:

We followed the reviewer's advice and further reorganized the manuscript to clearly separate experiments and interpretation.

(18) Try not to make a decisive argument unless solid evidence is available.

Authors:

We do not know exactly what the reviewer defines as 'solid evidence'. We have the impression that the reviewer means only validated experimental results, but validated thermodynamic theories are also solid evidence.

We provide:

(1) Experimental results (kinetics of vitrification of two metallic glasses) that are based on accurate analyses.

(2) A commonly used phenomenological description (VFTH equation) for which there is no fundamental theoretical framework.

(3) An interpretative model based on a validated thermodynamic approach (this is solid evidence).

(4) A comparison between model and results. This leads to a first explanation for the nonArrhenius behavior of the cooperative fluctuations based on experimental results. It is consistent with many published hypotheses and results from numerical simulations (also for metals), and is therefore a valid contribution to the current discussion.

HIGHLIGHTED VERSION OF THE REVISED MANUSCRIPT.

The cooperativity of atomic fluctuations in highly supercooled glass-forming metallic melts

Jürgen E. K. Schawe[1], Min Kyung Kwak[1,2], Mihai Stoica[1], Eun Soo Park[2], Jörg F. Löffler[1]*

[1] Laboratory of Metal Physics and Technology, Department of Materials, ETH Zurich, 8093 Zurich, Switzerland

[2] Department of Materials Science and Engineering, Research Institute of Advanced Materials & Institute of Engineering Research, Seoul National University, Seoul 08826, Republic of Korea

Corresponding Author

*juergen.schawe@mat.ethz.ch
ABSTRACT. The behavior of supercooled glass-forming metals depends on the cooperative atomic fluctuations caused by dynamic heterogeneities in the melt. These spatial and temporal heterogeneities form dynamic clusters, which are regions of cooperative rearrangement (CRR). In this study, the macroscopic kinetics and the correlation length ξ, of the CRR, are derived for $Pt_{57.4}Cu_{14.7}Ni_{5.3}P_{22.6}$ and $Pd_{43}Cu_{27}Ni_{10}P_{20}$ metallic glass-formers by fast differential scanning calorimetry near the glass transition. While the alloy composition influences the α-relaxation and vitrification kinetics, typically defined by the glass transition, as well as the limiting temperature of the Vogel-Fulcher-Tammann-Hesse equation and the fragility index, it has no significant influence on the correlation length of the cooperative atomic motions. In agreement with many other materials, ξ is about 3 nm at the glass transition for both metallic glasses. The temperature dependence of ξ correlates with the apparent activation energy of the α-relaxation and is the reason for its non-Arrhenius behavior.

TOC GRAPHICS

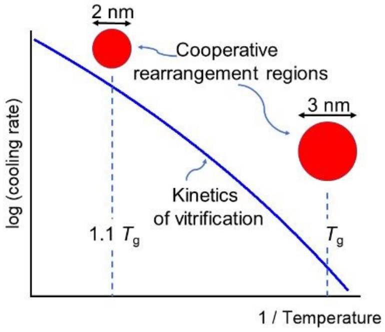

KEYWORDS metallic glasses; glass transition; dynamic heterogeneities; supercooled melt; cooperative rearrangements.

A metallic glass is formed if the melt is cooled rapidly enough to prevent crystallization. The critical cooling rate, $\beta_{c,crit}$, is the minimum rate at which such amorphous metal is created. This property can be used to characterize the glass-forming ability (GFA) of a metallic alloy. [1,2] Glass formation is favored when the atomic mobility in the supercooled liquid is relatively low and the viscosity is high: in glass-forming alloys, the latter is usually about three orders of magnitude higher than in pure metals that do not form a glass.[3] The nanostructure in supercooled glassforming melts is characterized by the formation of temporally and spatially fluctuating dynamic heterogeneities,[4,5] which are key to understanding the glass-forming kinetics, and, subsequently, the macroscopic properties of such materials.

According Based on to the the von Laue thermodynamics of fluctuations[6] classical thermodynamic theory of supercooled melts by Adam and Gibbs developed the classical theory of supercooled melt in that, the dynamic heterogeneities are governed by characteristic thermodynamic subsystems, i.e.- cooperative rearrangement regions (CRR) that determine the related configurational entropy.[7] Many dynamic effects in glass-forming melts can be explained using this classical approach.[8,9,10] The macroscopic time-temperature relation of the CRR fluctuations characterizes the α-relaxation. This is the relaxation process associated with the largest change in mechanical properties, where the viscosity changes from liquid to solid.

The dynamics of the glass transitionthe α relaxation in the supercooled melt is studied by using temperature modulated calorimetry and measurement of the vitrification kinetics in a wide cooling rate range on the example of two glass-forming metallic melts with different kinetics but sufficient stability against crystallization. These are the well-known $Pt_{57.4}Cu_{14.7}Ni_{5.3}P_{22.6}$[11] and $Pd_{43}Cu_{27}Ni_{10}P_{20}$.[12] metallic glasses. -From these experimental results and the temperature dependence of the correlation length of the CRR isare studied derived using thermodynamic approaches. on the example of two glass-forming metallic melts with different kinetics but sufficient stability against crystallization. These are the well-known $Pt_{57.4}Cu_{14.7}Ni_{5.3}P_{22.6}$[10] and $Pd_{43}Cu_{27}Ni_{10}P_{20}$.[11]

The kinetics of α-relaxation can be described as temperature dependence of the average relaxation time τ. Below the onset of cooperativity $\tau(T)$ follows the Vogel-Fulcher-TammannHesse (VFTH) equation:[13,14,15]

$$\log\left(\frac{1}{\tau}\right) = A - \frac{B}{T - T_\mathrm{v}} \qquad (1)$$

where A and B are fitting parameters, and T_v is the limiting temperature. The relaxation time is related to the measurement frequency according to $\tau\,\omega = 1$.

The thermodynamic definition of the glass transition temperature for calorimetric measurements is the limiting fictive temperature, T_f, according to Moynihan[16] and Richardson.[17]. It was used to determine all vitrification temperatures that were measured in the cooling rate range between 0.01 and 10,000 K s^{-1} in Fig. 1.

For thermo-rheological simple materials, i.e. materials with an almost temperature-invariant relaxation spectrum, the relaxation time is reciprocally proportional to the cooling rate, β_c, at which the material vitrifies at the same temperature:

$$(|\beta_\mathrm{c}|\,\tau)\cancel{/1\,K} = C \qquad (2)$$

where C is the proportionality constant.[18] Consequently, the relation between cooling rate and vitrification temperature also follows a VFTH equation with:

$$\log|\beta_\mathrm{c}| = A_\mathrm{c} - \frac{B}{T_\mathrm{f} - T_\mathrm{v}} \qquad (3)$$

where T_f is the fictive temperature, and B and $A_\mathrm{c} = A + \log C$ are constants.

Another frequently discussed kinetic parameter of the glass transition is the fragility index, m, which was introduced by Angell:[19]

$$m = \left(\frac{\mathrm{d}\log|\beta_\mathrm{c}|}{\mathrm{d}\frac{T_\mathrm{g}}{T}}\right)_{T=T_\mathrm{g}} = \frac{B\,T_\mathrm{g}}{(T_\mathrm{g} - T_\mathrm{v})^2} \qquad (4)$$

where T_g is the glass transition temperature after cooling the liquid at 10 K min^{-1}. Minor differences in the fragility index between relaxation and vitrification are due to different definitions of T_g and/or insufficient consideration of the influence of thermal inertia in the calorimeter.

The cooling rate dependence of T_f was measured in two ways. First, the samples were cooled at a cooling rate β_c from the supercooled liquid to the glassy state. Subsequently, they were heated into the melt regime at a heating rate $\beta_\mathrm{h} = |\beta_\mathrm{c}|$.[20] The T_f determined during heating and cooling must be identical, and deviations are due to uncertainties in determination and thermal inertias of the device and sample during the measurements. Fig. 1 shows that the measured T_f values upon

cooling and heating differ at rates above 10 K min^{-1} for conventional DSC and 200 K s^{-1} for FDSC. Above these scanning rates, the measurements must be corrected for thermal lag, for example by using the Tian equation:[21]

$$\Phi = \Phi_\mathrm{m} + \tau_\mathrm{in} \frac{\mathrm{d}\,\Phi_\mathrm{m}}{\mathrm{d}t} \quad (5)$$

where Φ and Φ_m are the corrected and measured heat flow, and τ_in is the time constant of the thermal inertia, which is determined from the difference of the measured fictive temperatures of the heating and cooling measurements, $\Delta T_\mathrm{f} = T_\mathrm{f,h} - T_\mathrm{fc}$:

$$\tau_\mathrm{in} = \frac{\Delta T_\mathrm{f}}{2\beta_\mathrm{h}} \quad (6)$$

The corrected fictive temperature is the mean value between $T_\mathrm{f,h}$ and $T_\mathrm{f,c}$.[18] These measurements were performed upon heating and cooling with rates between 2 and 40 K min^{-1} for DSC and 100 and 5000 K s^{-1} for FDSC. To close the gap between the two devices, additional measurements were performed using cooling rates between 1 and 50 K min^{-1} (with DSC) and 1 and 500 K s^{-1} (with FDSC). The subsequent heating rate was 30 K min^{-1} (DSC) or 1000 K s^{-1} (FDSC). The fictive temperatures were determined from the heating measurements and corrected according to the thermal inertia. In the respective Arrhenius diagram, the logarithm of the cooling rate is plotted against the reciprocal of the limiting fictive temperature T_f (Fig. 2). The parameters of the VFTH equation are determined by best fit. The glass transition temperature is T_f at a cooling rate of 10 K/min and the fragility index was calculated from the parameters according to Eq. (4).
These values are listed in Table 1.

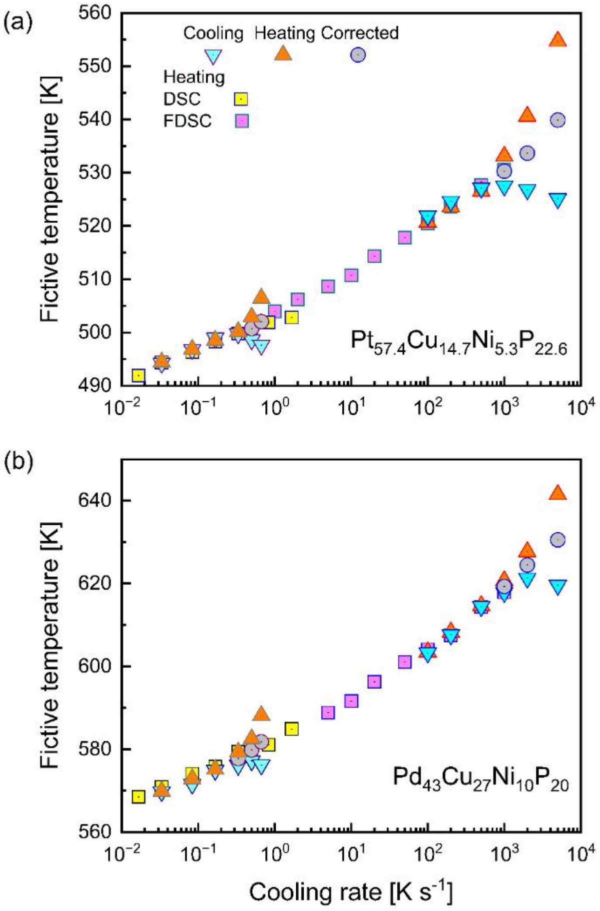

Figure 1. Limiting fictive temperature as a function of the cooling rate or the previous cooling rate for the heating measurements. The triangles are measured upon cooling (downward triangles) and subsequent heating at $\beta_h = -\beta_c$ (upward triangles), the circles are corrected data, and the squares are data measured at heating with a constant rate of 30 K min^{-1} (yellow squares) or 1000 K s^{-1} (pink squares) after cooling at various rates as indicated by the abscissa. (a) $Pt_{57.4}Cu_{14.7}Ni_{5.3}P_{22.6}$, (b) $Pd_{43}Cu_{27}Ni_{10}P_{20}$.

Table 1: Characteristic properties obtained from the Arrhenius diagrams in Figure 2.

Material	T_g [K]	A_c	B [K]	T_v [K]	m	$\xi(T_g)$ [nm]	$\frac{dE_a}{d\xi}$ [kJ mol^{-1} nm^{-1}]
$Pt_{57.4}Cu_{14.7}Ni_{5.3}P_{22.6}$	498	13.7	1310	407.9	80	2.80	376
$Pd_{43}Cu_{27}Ni_{10}P_{20}$	576	12.9	1534	463.2	69	2.65	350

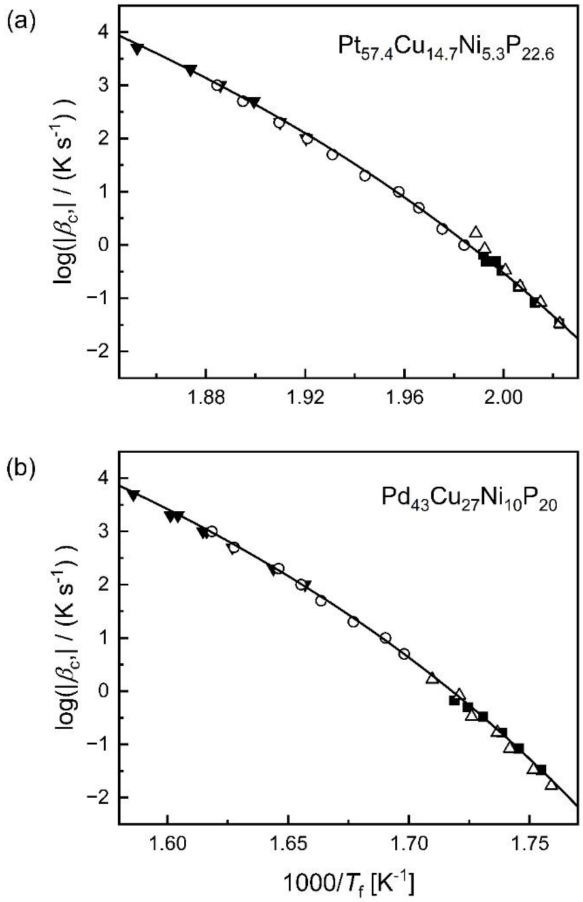

Figure 2. Arrhenius diagrams. The filled symbols are data measured at $\beta_c = -\beta_h$, while the empty symbols correspond to data measured at a constant heating rate (circles at 1000 K s^{-1} and triangles at 0.5 K s^{-1}). (a) $Pt_{57.4}Cu_{14.7}Ni_{5.3}P_{22.6}$, (b) $Pd_{43}Cu_{27}Ni_{10}P_{20}$.

A frequently discussed kinetic parameter is the width of the relaxation spectrum, which describs the distribution of relaxation times. For the α relaxation, the temperature width ΔT_ω, which is measured by modulated calorimetry at the frequency ω, is directly related to the width of the relaxation spectrum $\Delta \ln \omega$.[18] From

$$\frac{\Delta ln\ \omega}{\Delta T_\omega} \approx -\frac{dln\ \omega}{dT} \quad (7)$$

with $\omega \approx 1/\tau$ we obtain from Eq. (1) for the relation between the width of temperature dispersion measurements and the corresponding results for frequency dispersion in thermo-rheological simple liquids.

$$\Delta T_{\omega} \approx -\frac{\Delta ln\,\omega\,(T-T_{v})^{2}}{ln 10\,B} \qquad (8)$$

T~~t~~he width of the relaxation transition ΔT_{ω}, ~~which~~ was measured using temperature-modulated calorimetry.[22] The ΔT_{ω} were obtained from stochastic modulation (TOPEM) at 10 mHz in DSC and the temperature-modulated FDSC curves at 5 Hz, and correspond to the inflection temperature of the complex heat capacity curve, $|c_p^*|(T)$.[23] The width of the vitrification process during cooling, ΔT_{β}, is larger than ΔT_{ω}. This effect is described by the vitrification function $\kappa = \Delta T_{\beta} / \Delta T_{\omega}$,[18] where κ is a constant in the validity range of the VFTH equation.[18,24] For the investigated materials, κ was determined as 2.2 for $Pt_{57.4}Cu_{14.7}Ni_{5.3}P_{22.6}$ and 2.3 for $Pd_{43}Cu_{27}Ni_{10}P_{20}$. Typical cooling cures for $Pd_{43}Cu_{27}Ni_{10}P_{20}$ and the related evaluation of ΔT_{β} are shown in Fig. 3a. As expected, the temperature width increases with increasing temperature of the α relaxation.

The ~~These characterizations of the~~ macroscopic kinetics of the supercooled melts ~~and the formation of glasses~~ can be used to analyze the cooperative rearrangements in the melt.

The concept of characteristic subsystems with temperature fluctuations in combination with linear response theory to describe the cooperative molecular rearrangement of α-relaxation in supercooled melts has been developed by Donth.[25,26,27] . This allows to calculate the average characteristic correlation length, ξ, of the CRR using the measured thermodynamic data:

$$\xi = 6\,k_B\left(\frac{T_f}{\pi}\,\frac{}{\rho}\left(\frac{}{\delta T}\right)^{2}\left(\frac{1}{c_{p,g}} - \frac{1}{c_{p,l}}\right)\right)^{1/3} \qquad (9)$$

where T_f is the characteristic temperature of vitrification, δT is the temperature fluctuation determined from the width of the glass transition, $c_{p,g}$ and $c_{p,l}$ are the specific heat capacities of the glassy and liquid state, ρ is the density, and k_B is the Boltzmann constant. This thermodynamic

approach is based on von Laue's thermodynamics of temperature fluctuations,[6] which was further developed by Landau and Lifshitz.[28] The validity of this approach has been recently verified by quasi-elastic neutron scattering for polymeric materials.[29] The dynamic heterogeneities slightly above the glass transition temperature were visualized Further validation for the applicability of this method is based on the visualization of dynamic heterogeneities in supercooled metal melts by electron correlation spectroscopy using the example of metallic glass nano-wires.[30] , and the their result agrees ment with Donth's model for CRR.[26] Furthermore, for organic materials, the correlation lengths determined by dielectric relaxation spectroscopy,[31,32] NMR[33] ,and light scattering[34] and direct measurements of the configurational entropy at low temperature[35] agree well with data derived from Donth's method.[36,37,24]

Although these methods have been applied to non-metallic glass-forming liquids, Donth's approach is based on a validated thermodynamic concept without assumptions about molecular or atomic structure and interaction. We can therefore also safely apply it to atomic systems. Differential scanning calorimetry (DSC), in conjunction with its fast chip-based version, allows to determine the correlation length over a wide temperature interval by studying vitrification over a large cooling-rate range.[24,22,38]

While it is likely that cooperative spatial fluctuations in highly supercooled metallic melts affect the macroscopic behavior at the glass transition,[39] such as the mechanical properties[40] and the kinetics of glass formation and crystallization, there is limited information on the correlation length, ξ, and its temperature dependence.[41] Additionally, the relationship between ξ and the alloy composition remains unclear.

In a recent work,[24] the influence of structure on the correlation length of the CRR was investigated for two polymers and the chalcogenide glass-former selenium at nearly constant macroscopic kinetics. Here, we report for the first time the measurementscompare these results of $\xi(T)$ with measurements on two metallic glass-formers, which have distinct macroscopic kinetics.

The average temperature fluctuation in the CRR is δT.. can be derived from the width of the relaxation transition ΔT_ω, which was measured using temperature-modulated calorimetry.[31] The ΔT_ω were obtained from stochastic modulation (TOPEM) at 10 mHz in DSC and the temperature-modulated FDSC curves at 5 Hz, and correspond to the inflection temperature of the complex

heat capacity curve, c_p*(T).[36] The width of the vitrification process during cooling, ΔT_β, is larger than ΔT_ω. This effect is described by the vitrification function $\kappa = \Delta T_\beta / \Delta T_\omega$,[17] where κ is a constant in the validity range of the VFTH equation.[17,24x] For the investigated materials, κ was determined as 2.2 for $Pt_{57.4}Cu_{14.7}Ni_{5.3}P_{22.6}$ and 2.3 for $Pd_{43}Cu_{27}Ni_{10}P_{20}$. Typical cooling cures for $Pd_{43}Cu_{27}Ni_{10}P_{20}$ and the related evaluation of ΔT_β are shown in Fig. 3a.

For slowly crystallizing materials, the temperature dependence of the temperature fluctuation δT can be is determined directly from ΔT_ω.[22] Due to the higher crystallization tendency of metals, a larger temperature range can be achieved by scanning measurements. Therefore, the temperature fluctuation δT was calculated from the vitrification width ΔT_β of the (corrected) heat flow curves by:

$$\delta T = \frac{\Delta T_\beta}{2\,\kappa} \tag{10}$$

where the constant κ considers the broadening of the transformation interval during vitrification due to the slowing down of the change in relaxation time.[18]

Figure 3b reveals that the temperature fluctuation increases with increasing temperature.

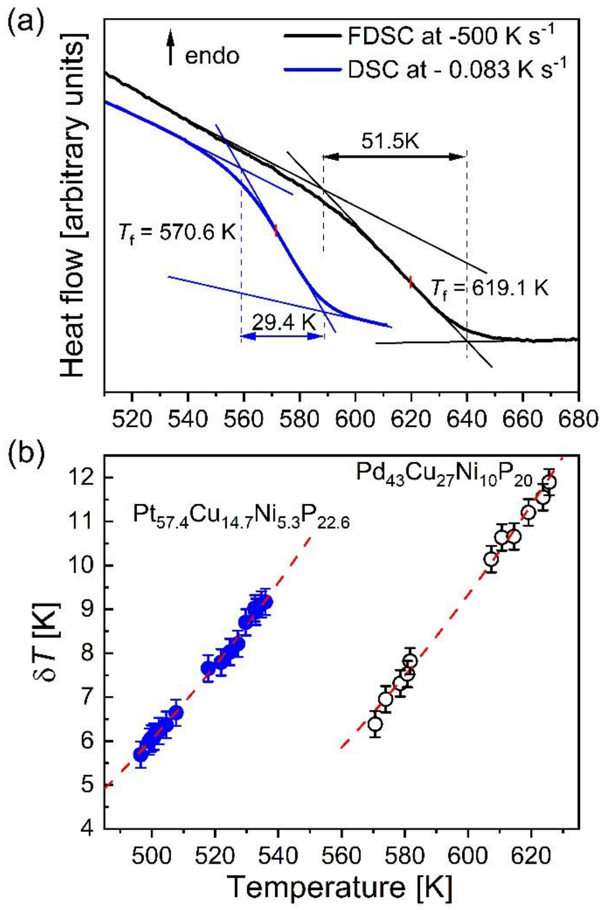

Figure 3. (a) Vitrification curves of $Pd_{43}Cu_{27}Ni_{10}P_{20}$ measured during cooling at -5 K min^{-1} (0.083 K s^{-1}) and -500 K s^{-1}, and evaluation of the limiting fictive temperature and width ΔT_β. (b) Average temperature fluctuation as a function of temperature. The dashed curves are guides for the eyes.

The specific heat capacities below and above the glass transition, $c_{p,\mathrm{g}}$ and $c_{p,\mathrm{l}}$, were measured using the TOPEM procedure. For the temperatures with known δT (see Fig. 3), the correlation length of the CRR was determined using Eq. (9). The values given in literature were used for the density.[11,12] The small change in ρ with temperature was neglected as it only has a minor influence on the uncertainty of ξ. The results are plotted in Fig. 4a, and it can be seen that ξ increases with decreasing temperature. The relative experimental uncertainty of ξ is approximately ±15 %. When comparing different methods and different materials, the absolute error is in the order of 0.5 nm.

At the glass transition temperature, ξ is just below 3 nm for both glass formers. The differences between the two materials of about 0.1 nm correspond to the order of experimental uncertainty. The correlation length is ~~measured~~ determined in a temperature interval of approximately $T_g \leq T \leq T_g$+50 K. In this range, ξ changes by ca. 50%, whereas the cooling rate and consequently the relaxation time (Eq. (2)) changes by about five orders of magnitude, as illustrated in Fig. 2. These two properties are thus not directly correlated, but it can be assumed that the change in size of the CRR is associated with the non-Arrhenius behavior of the dynamics in the supercooled melts. This can be expressed by an apparent activation energy for the relaxation process, E_a:

$$E_a(T) = -R \frac{d \ln|\beta_c|}{d\, 1/T_g} = \ln 10\, R \frac{B\, T^2}{(T - T_v)^2} \quad (11)$$

where R is the gas constant. Thus, the apparent activation energy in glass-forming melts increases with decreasing temperature. The concept of thermally activated cooperative rearrangements is widely accepted in literature.[42,43,44]

The comparison of the experimental results of ~~between~~ E_a and ξ in Fig. 4b indicates a linear relation, and thus suggests that the curvature in the Arrhenius diagram results from an increase in the size of the CRR. A more extensive discussion of the relation between E_a and ξ is given in the Supplementary Information.

The slope $\frac{d E_a}{d \xi}$ (listed in Table 1) is then a characteristic value that describes the increase in activation energy due to the increase in correlation length of the CRR. This value is expected to correlate with the fragility index m. However, the difference between the two slopes in Fig. 4b is insignificant for the two alloys investigated. The correlation between the increase of E_a and the growth of dynamic heterogeneities with temperature decrease, and ~~as well as~~ the insensitivity of the material ~~on~~ to the size of dynamic heterogeneities~~, was~~ were also observed in molecular dynamics simulations by the comparison of polymers and metals.[45]

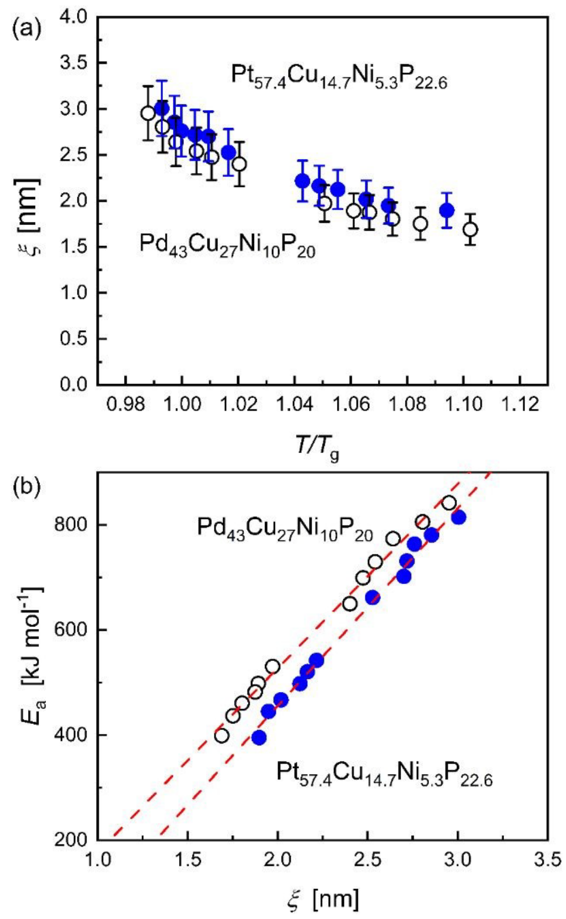

Figure 4. (a) Correlation length of the CRR as a function of reduced temperature T/T_g. (b) Apparent activation energy of the α-relaxation as a function of correlation length. The dashed lines are linear fits. The full symbols correspond to $Pt_{57.4}Cu_{14.7}Ni_{5.3}P_{22.6}$ and the open symbols to $Pd_{43}Cu_{27}Ni_{10}P_{20}$.

In conclusion, the measured macroscopic kinetics of the metallic melts and their vitrification a consistent with those of low-molecular organic materials and polymers. Therefore, the atomic (or molecular) origins for their macroscopic behavior are most likely comparable for different classes of materials. This is supported by the observation of dynamic heterogeneities in supercooled metallic melts close to vitrification.[30] By combining conventional DSC and FDSC, and after correcting the influence of thermal inertia, the activation curves of the vitrification process and the correlation length of the cooperative rearrangement regions (CRR) has been determined in a temperature range slightly below T_g and about 50 K above T_g for two glassforming alloys. The temperature range was wide enough to determine the macroscopic kinetic parameters (i.e., the Vogel-Fulcher-Tammann-Hesse (VFTH) parameters A, B and T_v) and the fragility parameter m. The correlation length ξ of the CRR increases from about 1.8 nm at $T/T_g \approx 1.1$ to ca. 3 nm at T_g. Surprisingly, no significant difference between the two materials has been found for the absolute

value of ξ and its dependency as a function of reduced temperature T/T_g, despite the fact that the kinetic parameters differ largely. This leads us to conclude that the compositional differences between the two glass-forming alloys are relevant for the macroscopic kinetic parameters but less important for the dimensions of the temporary heterogeneities associated with CRR. The increase in ξ with decreasing temperature appears to relate with the change in apparent activation energy of the cooperative atomic movements. From this we can draw three conclusions regarding the composition, the correlation length ξ, and the macroscopic kinetics:

(1) The composition and the related atomic interaction strongly influence the VFTH parameters and the glass transition temperature.

(2) The size of the dynamic fluctuations, relevant for the cooperative rearrangements near the glass transition, and its dependency on the reduced temperature T/T_g are not critically influenced by the composition.

(3) The increasing size of the spatial-temporal fluctuating domains with decreasing temperature correlates with the apparent activation energy of the relaxation process. This means that the increase in ξ with decreasing temperature is the reason for the nonArrhenius behavior of the α-relaxation.

EXPERIMENTAL METHODS

Cylindrical ingots of master alloys with a nominal composition of $Pd_{43}Cu_{27}Ni_{10}P_{20}$ and $Pt_{57.4}Cu_{14.7}Ni_{5.3}P_{22.6}$ (at.%)[46] were acquired from the PX Group, Switzerland. They were cut into small pieces, weighing around 4 grams, which were used to prepare amorphous ribbons via the melt spinning technique: the master alloy pieces were inductively melted in quartz crucibles, and then ejected through a nozzle of 1 mm diameter. The casting parameters were kept identical for both alloys. In detail, the nozzle-to-wheel distance was set to 0.3 mm, the ejection overpressure was 300 mbar and the Cu-wheel speed was 20 m/s. Although the melt spinning device is equipped with a two-color pyrometer, the actual ejection temperature could not be measured due to the low melting points of both alloys. The chamber was initially evacuated down to a pressure of 4 × 10^{-5} mbar, and for the melting and melt spinning it was filled with Ar 6.0 purity (99.9999 %) up to a pressure of 500 mbar. The melt-spun ribbons were 1 mm wide and around 20 μm thick.

The densities of the two alloys were taken from literature, and are ρ = 15.020 g cm^{-3} for $Pt_{57.4}Cu_{14.7}Ni_{5.3}P_{22.6}$ [11] and ρ = 9.425 g cm^{-3} for $Pd_{43}Cu_{27}Ni_{10}P_{20}$.[12]

Conventional DSC measurements were performed using a DSC823^e Mettler-Toledo device equipped with a sample robot and cryostat. The ribbons were cut into small sections and placed in an oxidized Al crucible. The cell gas was argon and the protective gas was nitrogen. The typical sample size was 60 mg. The temperature range for the Pt-based alloy was between 170 and 275 °C, and for the Pd-based alloy it was between 220 and 350 °C.

Temperature-modulated DSC measurements were performed using the stochastic modulation mode (TOPEM)[47] of the DSC823^e device. The measurements were carried out during cooling with a rate of 1 K min^{-1}. The measurement parameters are a temperature amplitude of 1 K and switching times of 30 and 31 s. The evaluation parameters relate to a calculation window of 200 s, a sample response parameter of 20, and an instrumental response parameter of 45. These measurements were performed to determine the heat capacity, and the heat capacity relaxation at 10 mHz.

A Flash DSC 2+ (Mettler-Toledo) with an IntraCooler TC100 (Huber) and a UFH 1 sensor were used for fast DSC (FDSC) measurements. The cell gas was Ar. A thin oil film was prepared on the sample side before inserting the sample with a typical weight of 1 μg. The temperature ranges were 25 °C $\leq T \leq$ 320 °C and 25 °C $\leq T \leq$ 440°C, respectively. Temperature-modulated measurements at 5 Hz were carried out with a saw-tooth modulation during cooling from the supercooled melt with an amplitude of 1 K and an underlying cooling rate of 2 K/s (further details in Ref. 48).

AUTHOR INFORMATION

Corresponding Author

Jürgen E.K. Schawe – *Laboratory of Metal Physics and Technology, Department of Materials, ETH Zurich, 8093 Zurich, Switzerland;*

Email: juergen.schawe@mat.ethz.ch

Authors

Min Kyung Kwak - *Laboratory of Metal Physics and Technology, Department of Materials, ETH Zurich, 8093 Zurich, Switzerland; Department of Materials Science and Engineering, Research Institute of Advanced Materials & Institute of Engineering Research, Seoul National University, Seoul 08826, Republic of Korea*

Mihai Stoica - *Laboratory of Metal Physics and Technology, Department of Materials, ETH Zurich, 8093 Zurich, Switzerland*

Eun Soo Park - *Department of Materials Science and Engineering, Research Institute of Advanced Materials & Institute of Engineering Research, Seoul National University, Seoul 08826, Republic of Korea*

Jörg F. Löffler - *Laboratory of Metal Physics and Technology, Department of Materials, ETH Zurich, 8093 Zurich, Switzerland*

AUTHOR CONTRIBUTIONS

J.E.K.S.: Conceptualization, Methodology, Investigation, Writing original draft. M.K.K.: Formal analysis, Investigation, Writing review and editing. M.S.: Investigation, Writing original draft. E.S.P.: Funding acquisition, Writing review and editing. J.F.L.: Conceptualization, Resources, Project administration, Writing review and editing.

ACKNOWLEDGEMENTS

The authors gratefully acknowledge the support of the ETH+ initiative within the framework of SynMatLab (Laboratory for Multiscale Materials Synthesis and Hands-On Education). ESP and MKK were supported by the Creative Materials Discovery Program through the National Research Foundation of Korea (NRF) funded by the Ministry of Science and ICT (No. NRF2019M3D1A1079215).

REFERENCES

(1) Löffler, J. F.; Schroers, J.; Johnson, W. L. Time-temperature-transformation diagram and microstructures of bulk glass forming Pd40Cu30Ni10P20. *Appl. Phys. Lett.* **2000**, *77*, 681–683.

(2) Shen, Y.; Perepezko, J. H. Al-based amorphous alloys: glass-forming ability, crys- tallization behavior and effects of minor alloying additions. *J. Alloys Comp.* **2017**, *707*, 3–11.

(3) Busch, R. The thermodynamical properties of bulk metallic glass-forming liquids, *JOM* **2000**, *52*, 39–42.

(4) Wang, X.; Xu, W.-S.; Zhang, H.; Douglas J. F. Universal Nature of Dynamic Heterogeneity in Glass-Forming Liquids: A Comparative Study of Metallic and Polymeric Glass-Forming Liquids. *J. Chem. Phys.* **2019**, *151*, 184503.

(5) Debenedetti, P. G.; Stillinger, F. H. Supercooled Liquids and the Glass Transition. *Nature* **2001**, *410*, 259–267.

(6) von Laue, M. Temperatur und Dichteschwankungen, *Physik. Z.* **1917**, *18*, 542-544.

(7) Adam, G.; Gibbs, H. J. On the Temperature Dependence of Cooperative Relaxation Properties in Glass-Forming Liquids. *J. Chem.Phys.* **1965**, *43*, 139–146.

(8) Berthier, L. Self-Induced Heterogeneity in Deeply Supercooled Liquids. *Phys. Rev. Lett.* **2021**, *127*, 088002.

(9) Starr, W. F.; Douglas, J. F.; Sastry, S. The relationship of dynamical heterogeneity to the Adam-Gibbs and random first-order theories of glass formation, *J. Chem. Phys.* **2013**, *138*, 12A541.

(10) Ozawa, M.; Scalliet, C.; Ninarello, A.; Berthier, L. Does the Adam-Gibbs relation holds in simulated supercooled liquids? *J. Chem. Phys.* **2019**, *151*, 084504.

(11) Gopinadhan, M.; Shao, Z.; Liu, Y.; Mukherjee, M.; Sekol, R. C.; Kumar, G.; Taylor, A. D.;

Schroers, J.; Osuji, C. O. Finite size effects in the crystallization of a bulk metallic glass. *Appl. Phys. Lett*. **2013**, *103*, 111912.

(12) Lu, I.-R.; Görler, G. P.; Fecht, H. J.; Willnecker, R. Investigation of specific volume of glass-forming Pd-Cu-Ni-P alloy in the liquid, vitreous and crystalline state. *J. Non-Cryst. Solids* **2002**, *312–314*, 547–551.

(13) Vogel, H. Temperaturabhängigkeitsgesetz der Viskosität von Flüssigkeiten. *Phys. Z.* **1921**, *22*, 645–646.

(14) Fulcher, G. S. Analysis of recent measurements of the viscosity of glasses. J. *Am. Ceram. Soc.* **1925**, *8*, 339–355.

(15) Tammann, G.; Hesse, W. Die Abhängigkeit der Viskosität von der Temperatur von unterkühlten Flüssigkeiten. *Z. Anorg. Allg. Chem*. **1926**, *156*, 245–257.

(16) Moynihan, C. T.; Easteal, A. J.; DeBolt, M. A.; Tucker, J. Dependence of the fictive temperature of glasses on cooling rate. *J. Am. Ceram. Soc.* **1976**, *59*, 12–16.

(17) Richardson, M. J.; Savill, N. G. Derivation of accurate glass transition temperatures by differential scanning calorimetry. *Polymer* **1975**, *16*, 753–757.

(18) Schawe, J. E. K. Vitrification in a wide cooling rate range: The relations between cooling rate, relaxation time, transition width, and fragility. *J. Chem. Phys.* **2014**, *141*, 184905.

(19) Angell, C. A. Spectroscopy Simulation and Scattering, and the Medium Range Order Problem in Glass. *J. Non-Cryst. Solids* **1985**, *73*, 1−17.

(20) Schawe, J. E. K. An analysis of the meta stable structure of poly(ethylene terephthalate) by conventional DSC. *Thermochim. Acta* **2007**, *461*, 145-152.

(21) Vyazovkin, S.; Sbirrazzuoli, N. Nonisothermal crystallization kinetics by DSC: Practical overview. *Processes* **2023**, *11*, 1438.

(22) Chua, Y. Z.; Schulz, G.; Shoifet, E.; Huth, H.; Zorn, R.; Schmelzer, J. W. P.; Schick, C. Glass transition cooperativity from broad band heat capacity spectroscopy. *Colloid Polym. Sci.* **2014**, *292*, 1893–1904.

(23) Schawe, J. E. K. Principles for the interpretation of modulated temperature DSC measurements. Part 1. Glass transition. *Thermochim. Acta* **1995**, *261*, 183-194.

(24) Hallavant, K.; Mejres, M.; Schawe, J. E. K.; Esposito, A.; Saiter-Fourcin, A. Influence of chemical composition and structure on the cooperative fluctuation in supercooled glassforming liquids. *J. Phys. Chem. Lett.* **2024**, *15*, 4508–4514.

(25) Donth, E. The size of cooperatively rearranging regions at the glass transition. *J. Non-Cryst. Solids* **1982**, *53*, 325-330.

(26) Donth, E. Phenomenological treatment of dynamic glass transition heterogeneity. *Acta Polym*. **1999**, *50*, 240 – 251.

(27) Donth, E. The glass transition, Springer-Verlag, Berlin, Heidelberg **2001**.

(28) Landau, L.D, Lifshitz, E.M, Statistical physics part 1, third edition, Pergamon Press, **1980.**

(29) Chua, Y. Z.; Zorn, R.; Schmelzer, J. W. P.; Schick, C.; Holderer, O.; Zamponi, M. Determination of Cooperativity Length in a Glass-Forming Polymer. *ACS Phys. Chem. Au*

2023, *3*, 172−180.

(30) Zhang, P.; Maldonis, J. J.; Liu, Z.; Schroers, J.; Voyles, P. M. Spatially heterogeneous dynamics in a metallic glass forming liquid imaged by electron correlation microscopy. *Nat. Comm*. **2018**, *9*, 1129.

(31) Cangialosi, D., Alegría, A.; Colmenero, J. Dielectric relaxation of polychlorinated biphenyl/toluenemixtures: Component dynamics. *J. Chem. Phys.* **2008**, *128*, 224508.

(32) Berthier, L.; Biroli, G.; Bouchaud, J.-P.; Cipelletti, L.; El Masri, D.; L'Hote, D.; Ladieu, F.; Pierno, M. Direct experimental evidence of a growing length scale accompanying the glass transition. *Science* **2005**, 310, 1797-1800.

(33) Qiu, X. H.; Ediger, M. D. Length scale of dynamic heterogeneity in supercooled Dsorbitol: comparison to model predictions. *J. Phys. Chem. B* **2003**, *107*, 459-464.

(34) Hong, L., Novikov, V.N.; Sokolov, A.P. Dynamic heterogeneities, boson peak, and activation volume in glass-forming liquids. *Phys. Rev E* **2011**, *83*, 061508.

(35) Tatsumi, S.; Aso, S.; Yamamuro, O. Thermodynamic study of simple molecular glasses: Universal features in their heat capacity and the size of the cooperatively rearranging regions. *Phys. Rev. Lett.* **2012**, *109*, 045701.

(36) Hempel, E.; Hempel, G.; Hensel, A.; Schick, C.; Donth, E. Characteristic length of dynamic glass transition near Tg for a wide assortment of glass-forming substances. *J. Phys. Chem. B* **2000**, *104*, 2460-2466.

(37) Rijal, B., Delbreilh, L.; Saiter, A. Dynamic Heterogeneity and Cooperative Length Scale at Dynamic Glass Transition in Glass Forming Liquids. *Macromolecules* **2015**, *48*, 8219– 8231.

(38) Schawe, J. E. K. Measurement of the thermal glass transition of polystyrene in a cooling rate range of more than six decades. *Thermochim. Acta* **2015**, *603*, 128-134.

(39) Wei, S.; Everson, Z.; Galano, I.; Busch, R. The impact of fragility on the calorimetric glass transition in bulk metallic glass. *Intermetallics* **2014**, *55*, 138-144.

(40) Jiang, M .Q.; Wilde, G.; Jiang, F.; Dai, L. H. Understanding ductile-to-brittle transition of metallic glasses from shear transformation zone dilatation. *Theoretical and Applied Mechanics Letters*, **2015**, *5*, 200-204.

(41) Wang, T.; Li, J. B.; Yang, J. Y.; Rao, G. H. A comparative study on characteristic relaxation in $Ce_{65}Al_{10}Cu_{20}Co_5$ and $Zr_{46.75}Ti_{8.25}Cu_{7.5}Ni_{10}Be_{27.5}$ bulk metallic glasses and supercooled liquids. *Intermetallics* **2011**, *19*, 81-85.

(42) Annamareddy A.; Voyles, P. M.; Perepezko, J.; Morgan, D. Mechanisms of bulk and surface diffusion in metallic glasses determined from molecular dynamics simulations, *Acta Mater*. **2021**, *209*, 116794

(43) Liu, C.; Pineda, E.; Crespo, D. Mechanical relaxation of metallic glasses: An overview of experimental data and theoretical models. *Metals* **2015**, *5*, 1073-1111.

(44) J.M. Pelletier, J. M.; Cardinal, S.; Qiao, J. C.; Eisenbart, M.; Klotz, U. E. Main and secondary relaxations in an Au-based bulk metallic glass investigated by mechanical spectroscopy. *J. Alloys Compd*. **2016**, *684*, 530-536

(45) Zhang, H.; Wang, X.; Yu, H.-B.; Douglas, J. F. Dynamic heterogeneity, cooperative motion, and Johari-Goldstein β-relaxation in a metallic glass-forming material exhibiting a fragile-to-strong transition. *Eur. Phys. J. E* **2021**, *44*, 56.

(46) Haag, F.; Sauget, R.; Kurtuldu, G.; Prades-Rödel, S.; Schawe, J. E. K.; Blatter, A.; Löffler, J. F. Assessing continuous casting of precious bulk metallic glasses. *J. Non-Cryst. Solids* **2019**, *521*, 119120.

(47) Schawe, J. E. K.; Hütter, T.; Heitz, C.; Alig, I.; Lellinger, D. Stochastic temperature modulation: A new technique in temperature-modulated DSC. *Thermochim. Acta* **2006**, *446*, 147-155.

(48) Lindemann, N.; Schawe, J. E. K.; Lacayo-Pineda, J. Kinetics of the glass transition of silica-filled styrene-butadiene-rubber: The effect of resins. *Polymers* **2022**, *14*, 2626.
